# Development of plasma and whole blood taurine reference ranges and identification of dietary features associated with taurine deficiency and dilated cardiomyopathy in golden retrievers: A prospective, observational study

**DOI:** 10.1371/journal.pone.0233206

**Published:** 2020-05-15

**Authors:** Eric S. Ontiveros, Bradley D. Whelchel, Joshua Yu, Joanna L. Kaplan, Ashley N. Sharpe, Samantha L. Fousse, Amanda E. Crofton, Andrea J. Fascetti, Joshua A. Stern

**Affiliations:** 1 Department of Medicine and Epidemiology, School of Veterinary Medicine, University of California Davis, Davis, CA, United States of America; 2 Department of Molecular Biosciences, School of Veterinary Medicine, University of California Davis, Davis, CA, United States of America; University of Bari, ITALY

## Abstract

**Introduction:**

A surge in Food and Drug Administration (FDA) consumer complaints identified concerns that legume-rich, grain-free diets were associated with nutritionally-mediated dilated cardiomyopathy (DCM). Golden retrievers represent the most reported breed affected by this condition and previous studies documented the disease is responsive to dietary change and taurine supplementation. Although dietary findings across cases are compelling, prospective studies with control groups are lacking. The role of diet in developing taurine deficiency and echocardiographic changes consistent with DCM in healthy dogs is unknown.

**Objectives:**

We hypothesized that golden retrievers eating non-traditional diets are at a higher risk of having taurine deficiency and nutritionally-mediated DCM compared with those eating traditional commercial diets. We aimed to compare taurine concentrations and echocardiographic indices of systolic function between golden retrievers in each diet group and elucidate associations between diet and these variables. Additionally, we aimed to generate breed-specific reference intervals for whole blood and plasma taurine concentrations.

**Animals:**

86 golden retrievers.

**Methods:**

Golden retrievers eating traditional or non-traditional diets were evaluated and diet history, taurine concentrations and echocardiographic data were collected. Dietary features, taurine concentrations and echocardiographic findings were compared between diet groups. Relative risks were calculated for the likelihood of echocardiographic abnormalities and taurine deficiency in each diet group. Breed-specific reference intervals were constructed for taurine concentrations in dogs from the traditional diet group.

**Results:**

Golden retrievers eating non-traditional diets had significantly lower taurine concentrations and more frequent systolic dysfunction. Breed specific reference intervals are higher than previously reported across breeds.

**Conclusions:**

Non-traditional diets, which were typically grain-free and contained legumes in this study, were significantly associated with and have increased relative risk for the identification of taurine deficiency and echocardiographic abnormalities consistent with nutritionally-mediated DCM. These findings were identifiable in the absence of clinical signs and support the findings of multiple previous studies and the ongoing FDA investigation.

## Introduction

Canine dilated cardiomyopathy (DCM) represents the second most common acquired heart disease in dogs and has multiple identified etiologies [1–3]. While DCM of genetic origins have been described for some breeds based upon discovered mutations or observed heritability and pattern of inheritance, determining etiology of DCM when observed outside of these breeds is challenging [4–16]. Nutritionally mediated DCM has been described across a variety of species including dogs and is most historically linked to taurine deficiency [17–27]. Recent peer-reviewed research on DCM in breeds that were not previously known to have a genetic etiology has raised concern about the relationship between diets with certain characteristics and the development of nutritionally-mediated DCM [28–30]. The Food and Drug Administration issued a warning and subsequently released data that identified dietary characteristics which were over-represented in consumer concern reports [31,32]. This data is supported by similar findings from researchers at multiple institutions and suggests that diets which are grain-free, contain legume or potato ingredients warrant study to further elucidate a possible role in the causation of DCM [28,29]. When evaluated, the FDA data also identifies an inverse relationship/correlation between the size of a company in terms of worldwide sales and the number of reported cases of DCM where smaller companies have the highest reported case numbers [32].

Of all breeds represented in the research and FDA report, the golden retriever is consistently the most frequently reported breed to be affected by nutritionally-mediated DCM [20,22,23,29,32]. The role of taurine deficiency in this breed appears more relevant when compared to other breeds eating similar diets [20,22,23,29]. The over-representation of golden retrievers is interesting as there is no literature to support any familial relationship or genetic etiology for classic DCM in golden retrievers. In fact, results of a large golden retriever breed club survey in 1999 showed the incidence of cardiomyopathy in >1400 golden retrievers to be less than 0.7% with no further classification information provided [33]. Additionally, a large study of heart disease of insured dogs identified golden retrievers as a low risk breed for all cardiac claims and having a lower cardiac mortality rate than the pooled study population (8 compared to 22 respectively)[34]. Numerous reports have implicated that golden retrievers may be more susceptible to taurine deficiencies [20,22,23,29]. Thus, the investigators sought to study the relationship between diet and dietary ingredients with nutritionally-mediated DCM with or without taurine deficiency. The authors have previously reported on 24 cases of nutritionally-mediated DCM in golden retrievers and their response to therapy. This previous study demonstrated that dogs of this breed with clinical disease have a high likelihood of disease reversal when diet change and taurine supplementation is implemented [29]. However, this previous study was a prospective clinical case series that followed patients over time and introduced new questions about the incidence of this disease in other populations and the role of diet and dietary ingredients in this disease process [29].

The objective of this study was to evaluate the incidence of nutritionally-mediated dilated cardiomyopathy in a population of healthy golden retrievers presented for breed screening evaluations. We hypothesized that golden retrievers eating what we defined as non-traditional commercial diets are at a higher risk of having taurine deficiency and nutritionally-mediated DCM than those eating what we defined as traditional commercial diets. We aimed to compare taurine concentrations in plasma and whole blood, echocardiographic indices of heart size and systolic function between golden retrievers in each diet group and elucidate any associations between diet group and these variables. Additionally, we aimed to compare reference intervals generated from the traditional diet group to those historically used from previous multi-breed studies.

## Materials & methods

Golden retrievers were recruited for this prospective observation study from the populations of the cardiovascular breed screening examinations at the Cardiac Genetics Research Laboratory of the University of California Davis, breed screening events hosted by breed clubs or in response to the study advertisement on the university clinical trials webpage between the years of 2017 and 2019. Individuals identified as possible participants through one of these routes were offered the opportunity to participate in this research. The study was approved by the Institutional Animal Care and Use Committee of the University of California Davis and all study participants provided informed consent. Inclusion criteria required the dogs be free of any reported clinical signs of disease, have an unchanged diet history for at least 3 months, allow complete echocardiographic examination without the requirement for sedation and provide a complete diet history.

### Treatment groups and diet history

Diet groups were defined and those not meeting either category were not enrolled in the study, but rather continued as clinical patients. Traditional diets (TD) were required to meet all of the following criteria: kibble diets which are grain-inclusive, not including legumes or potatoes in the top 5 ingredients listed and be produced by a pet food company with >$2billion in global sales for 2018. Non-traditional diets (NTD) had to meet one of the following criteria: kibble or raw food diet which is grain free, includes legumes or potatoes in the ingredient list, or is manufactured by a small pet food company with <$1billion in global sales for 2018. Publicly available global sales information of the parent company was used as previously reported [35,36]. These categories were defined a priori based upon reported FDA data and the authors clinical experience showing overlapping results that the disease is most frequently observed in dogs fed grain free diets with legumes or potatoes often in the top 5 positions of the ingredient list [32]. It is further supported by the FDA data and the authors clinical experience that there is a relative absence of cases from large companies, particularly when compared to market share of the individual diets, where the most abundant cases reported were from companies with very small market share [32,36,37].

Enrollment targeted at least 40 dogs in each group to provide the ability to meet American Society for Veterinary Clinical Pathology (ASVCP) guidelines for the generation of reference intervals, thus once 40 dogs were met in each group the study enrollment was stopped [38]. Body weight (kg) and sex and whether the dog was castrated, spayed or intact was recorded. Body condition score was assessed by the attending veterinary cardiologist and recorded using a validated 9-point scale [39]. Muscle condition score was assessed by the attending veterinary cardiologist and recorded using a 4 point scale [40]. A standardized diet history form (https://ccah.vetmed.ucdavis.edu/sites/g/files/dgvnsk4586/files/inline-files/Study_Related_Diet%20History_Form.pdf) was utilized for this study and verified for completeness prior to enrollment. When information was unclear at the time of data entry, the owners were contacted to provide additional details as needed. Data collected included brand names and varieties of the diet(s) fed at the time of sample collection. Daily quantity fed and length of time (months) the dog had been fed the same diet regimen was recorded. All medications, supplements or probiotics the dog was receiving were recorded. Dogs with diet histories not meeting one of the defined categories were excluded (i.e. dogs eating a mix of diets from the TD and NTD group, etc.). Dogs with incomplete diet histories that could not be elucidated through owner contact were excluded. Dogs that did not have a consistent diet history for >3 months were excluded. Dogs receiving supplements containing taurine, methionine or l-carnitine were excluded. Dogs receiving non-commercial foods that accounted for >10% of their diet were excluded. Dogs receiving canned pumpkin as a method to increase dietary fiber were excluded.

For each diet, the listed ingredients and calorie content (kcal/cup and kcal/kg) when available were reviewed and recorded. Diet data was recorded using archived website data within 6 months of the date of study enrollment when possible [41]. When archived web data was not available, the diet data was obtained from the current internet listed product details at the time of data analysis (July 2019). For each diet, the inclusion of legumes or potatoes was recorded in several ways: 1) are any legume or potato ingredients within the top 5 ingredients listed, 2) are any legumes or potatoes in the diet ingredient list, 3) the total number of legumes or potatoes found in the ingredient list. Whether or not the diet was reported to be grain free was recorded and verified by review of the ingredient list. The inclusion of taurine or methionine in the ingredient list was recorded. Supplements reportedly administered were reviewed for the inclusion of taurine or methionine. If supplements were found to contain taurine or methionine the dog was excluded from the study.

Resting and maintenance energy requirements were calculated as previously reported [29]. Resting energy requirement (RER) was calculated for each dog using the following equation: RER = 70 x BW^0.75^, where BW represents body weight in kilograms [42]. Maintenance energy requirement (MER) was calculated as a range to provide the requirement for a sedentary to more active lifestyle [42]. The MER calculation was performed by multiplying RER by 1.4 to 1.6 and providing the resulting range [42]. Percent differences between the amount fed in kcal/day and the calculated MER range were calculated.

### Blood taurine concentration measurements

Venous blood samples were obtained for lithium heparinized whole blood taurine (always >1mL) and plasma taurine (when possible >2mL) concentration measurements. Fasting was not required prior to blood sampling and fasted or fed status was not recorded. Fasting status does not impact taurine status in dogs [43]. Samples were processed according to the guidelines of the Amino Acid Laboratory at UC Davis [44]. Plasma taurine samples were not obtained for some study participants where screening exams took place off site and expedient plasma preparation and storage was not possible. Plasma taurine concentrations <60nmol/mL or whole blood taurine concentrations <200nmol/mL were considered low according to published canine reference intervals [45,46].

### Echocardiography

All dogs received an echocardiogram by a board-certified veterinary cardiologist or resident in training under the direct supervision of a board-certified veterinary cardiologist and images were stored for off-line analysis. Off-line echocardiographic image analysis and measurements were performed by a single investigator who was blinded to diet history and assigned diet group (Syngo Dynamic Workplace, Version 10.0.01_HF04_Rev5 [Build 2884], Siemens Medical Solutions, Malvern Pennsylvania). Complete echocardiographic examinations were reviewed with the intent to exclude any patients from the study that had congenital cardiac disease capable of negatively impacting systolic function or increasing chamber size. Echocardiographic measures recorded for the study included measurement of left ventricular internal diameter in diastole (LVIDd), left ventricular internal diameter in systole (LVIDs) and calculated percent fractional shortening (%FS). Briefly left ventricular measures were performed from right parasternal short-axis m-mode imaging of the left ventricle at the level of papillary muscles and measures were performed and averaged from 3 consecutive sinus beats when possible avoiding the cycles immediately following any observed arrhythmias. Fractional shortening was calculated from the measures by the following equation: (LVIDd-LVIDs)/LVIDd x 100. Ejection fraction was calculated using the Teichholz method to determine chamber volumes as previously described [47]. End diastolic volume index and end-systolic volume index were also calculated by dividing the end-systolic or end-diatolic volumes in milliliters by body surface area in meters^2^ as previously described [48]. Body surface area was calculated by the formula 0.101 x body weight (kg)^2/3^. The fractional shortening was recorded as low when <25% [49,50]; LVIDd was considered increased when >51 mm [49], and LVIDs was considered increased when >35 mm [49]. These chosen cut-off values are based upon previously published data and breed specific reference intervals which also maintain consistency with prior publication[29,49,50].

### Statistical analysis

Statistical analysis was performed with the use of commercially available software (GraphPad Prism version 8.2.1 for macOS, GraphPad Software, San Diego, California USA, www.graphpad.com; MedCalc Statistical Software version 16.4.3, MedCalc Software bvba, Ostend, Belgium; www.medcalc.org; 2016). Data was inspected visually for normal distribution and tested for normality by D’Agostino-Pearson omnibus normality test. Column statistics were provided for all continuous variables with parametric data reported as mean +/- standard deviation (SD) and non-parametric data reported as median and interquartile range. When applicable, range was reported as minimum (min) and maximum (max) values. Differences between groups were tested for statistical significance by use of an unpaired t-test (when both data groups were parametric) or Mann-Whitney test (when either or both data groups were non-parametric). A P value less than 0.05 was considered significant.

Two-by-two contingency tables were constructed for evaluation of categorical variable outcomes such as low taurine concentration, elevated left ventricular chamber dimension (LVIDd or LVIDs) or reduced fractional shortening classifying no as 0 and yes as 1. Statistical associations between diet groups (TD vs. NTD) were tested using a Fishers exact test and the relative risk and 95% confidence interval of the relative risk of these outcomes are reported.

For all enrolled dogs where both plasma and whole blood taurine concentrations were available the data was evaluated for correlation (Spearman correlation). Significant correlation (P<0.05) was described as weak correlation (R2<0.2), moderate correlation (R2 = 0.2–0.4) or strong correlation (R2>0.4).

Reference intervals were established for dogs in the TD group according to the Clinical and Laboratory Standards Institute guidelines for determining reference values and reference intervals for quantitative clinical laboratory tests [51]. For normally distributed data, reference intervals were generated using commercially available software (MedCalc Statistical Software version 16.4.3, MedCalc Software bvba, Ostend, Belgium; www.medcalc.org; 2016) using the Robust method with 90% bootstrap confidence intervals of the reference limit reported and no outliers removed. For non-parametric data the reference intervals were generated using the nonparametric percentile technique with no outliers removed [51].

## Results

A total of 116 golden retrievers were considered for study inclusion. Twelve dogs were excluded for feeding a mix of TD and NTD. Twelve dogs were excluded for feeding a non-commercial food item that accounted for >10% of the daily calories in the diet. Four dogs were excluded for receiving supplements containing taurine methionine or l-carnitine. One dog was excluded for an inconsistent or unclear diet history over the 3 months preceding study enrollment. One dog was excluded for feeding food additives with intent of increasing dietary fiber (canned pumpkin). Eighty-six dogs remained after exclusions and were sorted into the TD (n = 43) and NTD (n = 43) groups. The investigators stopped study enrollment when the numbers met the predetermined level of >40 dogs per group.

The study was comprised of a total of 44 males (14 castrated) and 42 females (24 spayed). In the TD group there were 20 males (3 castrated) and 23 females (13 spayed). In the NTD group there were 24 male (11 castrated) and 19 females (11 spayed). The mean age +/- SD of the dogs was 4.1 +/-3.2 and 4.5 +/- 2.9 years respectively for the TD and NTD groups (P = 0.63). Body weight (mean +/- SD) was 28.9 +/- 4.9 and 31.1 +/- 4.7 for the TD and NTD groups respectively and was significantly higher in the NTD group (P = 0.04). Body condition score had a median of 5 (IQR 5, 6) in each group (P = 0.86). Muscle condition score was 4 for all enrolled dogs.

### Whole blood and plasma taurine concentrations

**Fig 1:**
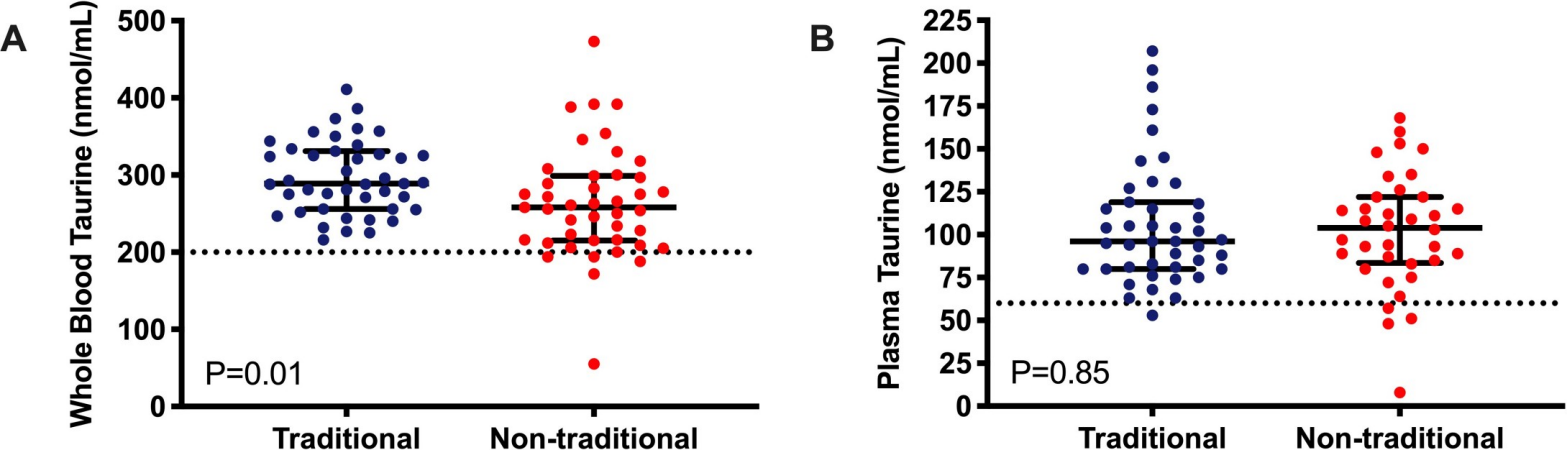
Differences in taurine concentrations for golden retrievers fed a traditional and non-traditional diet. A) Unpaired t-test results for whole-blood taurine concentration (nmol/mL). The dash line at 200 nmol/ml denotes the current threshold for low whole-blood taurine concentration. B) Mann-Whitney test results for plasma taurine concentration (nmol/mL). The dashed line at 60 nmol/mL denotes the current threshold for low plasma taurine concentration.

Whole blood taurine concentrations were available for all dogs in the study. None of the plasma samples were visibly hemolyzed prior to measuring taurine concentrations. Plasma taurine concentrations were available for all 43 dogs in the TD group and 36 dogs in the NTD group. Whole blood taurine concentrations were significantly lower in the NTD group (mean +/- SD; 264 +/- 72.0 nmol/mL; range 55–473 nmol/mL) when compared to the TD group (297 +/- 47.4 nmol/mL; range 216–411 nmol/mL; P = 0.01) (Fig 1A). Plasma taurine concentrations were not significantly different between groups with means of 106 +/- 36.2 nmol/mL in the TD group and 102.1 +/- 34.0 nmol/mL in the NTD group (P = 0.85) (Fig 1B).

### Echocardiographic data

**Fig 2:**
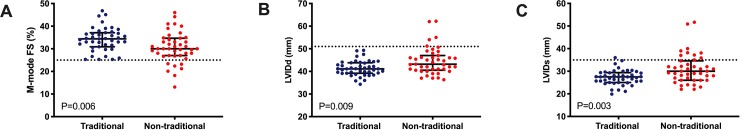
Results for echocardiographic variable differences for traditional and non-traditional diet groups. A) Unpaired t-test results for m-mode fractional shortening (FS) percent for different diet groups. The dashed line at 25% denotes the study threshold for a low FS. B) Mann-Whitney test results for left ventricular internal diameter in diastole (LVIDd) in mm for different diet groups. The dashed line at 51mm denotes the study threshold for diagnosing an increased LVIDd. C) Mann-Whitney test results for left ventricular internal diameter in systole (LVIDs) in mm for different diet groups. The dashed line at 35mm denotes the study threshold for diagnosing an increased LVIDs.

Fractional shortening was lower in the NTD group (30.6 +/- 6.7%; range 13.1–46.0%) compared to the TD group (34.2 +/- 5.2%; range 25.3–46.8%; P = 0.006; Fig 2A). Left ventricular diameter in diastole was significantly increased in the NTD group (median 43.2, IQR 36.3, 47 cm, range 36.3–62.2 cm) compared to the TD group (median 41.1, IQR 39.2, 43.8 cm; range 34.3–49.3 cm; P = 0.009; Fig 2B). Left ventricular diameter in systole was significantly increased in the NTD group (median 30.0, IQR 26.0, 34.6 cm, range 22.0–51.7 cm) compared to the TD group (median 27.5, IQR 25.0, 29.4 cm; range 19.9–36.0cm; P = 0.003; Fig 2C). Ejection fraction was significantly lower in the NTD group (58 +/- 9.9%; range 34–77%) compared to the TD group (63 +/- 7.1%; range 50–79%; P = 0.007; S1A Fig). End-systolic volume index was significantly higher in the NTD group (median 36.2, IQR 26.9, 48.3, range 16.8–118.4) compared to the TD group (median 29.5, IQR 24.4, 34.2, range 13.1–62.4; P = 0.013; S1B Fig). End-diastolic volume index was not significantly different between the NTD group (median 84.3, IQR 77.2, 102.8, range 55.1–179.8) when compared to the TD group (median 79.9, IQR 73.0, 86.9, range 50.3–129.9; P = 0.061; S1C Fig).

### Relative risk

**Fig 3:**
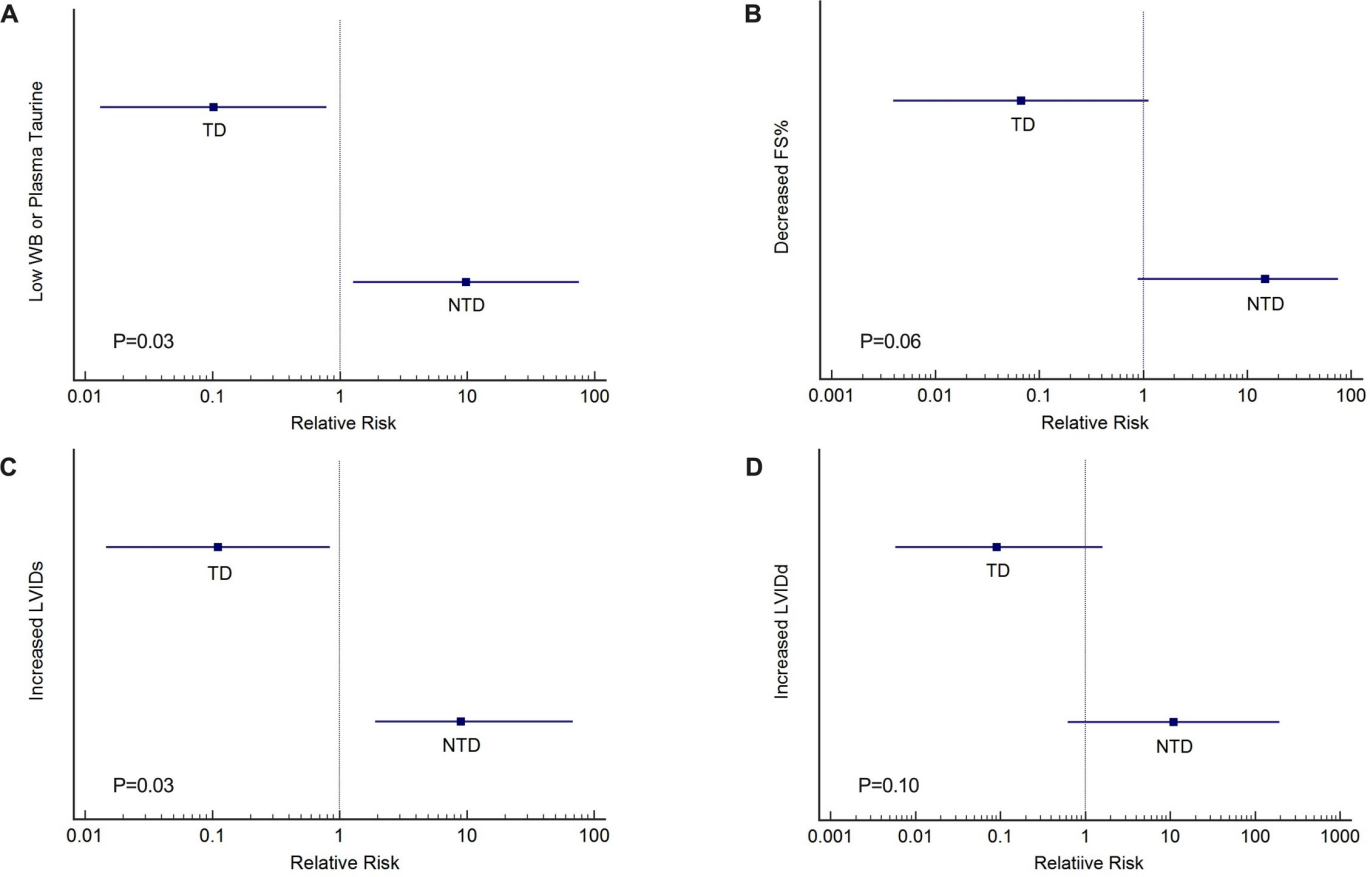
Forest plots illustrating the relative risk for consuming a traditional (TD) and non-traditional (NTD) diet for A) low whole-blood or plasma taurine, B) decreased fractional shortening (FS) percent, C) increased left ventricular internal diameter in systole (LVIDs), and D) increased left ventricular internal diameter in diastole (LVIDd).

Fisher’s exact testing identified that total instances of low taurine (either whole blood or plasma) were associated with feeding a NTD diet (P = 0.007). There were 9 instances of low whole blood or plasma taurine concentration in the NTD group compared to 1 in the TD group and 70 and 85 normal taurine concentration instances respectively resulting in a relative risk of 9.8 (95% CI 1.3–75.6, z statistic 2.2, P = 0.03) (Fig 3A). Diet characteristics for the TD and NTD groups are listed in Tables 1 and 2 respectively. Fisher’s exact testing identified low FS% was associated with feeding a NTD diet (P = 0.007). There were 7 instances of low FS% in the NTD group and 0 instances in the TD group with 36 and 43 normal FS% cases respectively resulting in a relative risk of 15.0 (95% CI 0.88–254.7, z statistic 1.87, P = 0.06) (Fig 3B). Fisher’s exact testing identified that an elevated LVIDs was associated with the NTD diet group (P = 0.01). There were 9 instances of increased LVIDs in the NTD group and 1 instance in the TD group with 34 and 42 normal LVIDs measurements respectively resulting in a relative risk of 9.0 (95% CI 1.2–68.0, z statistic 2.13, P = 0.03) (Fig 3C). Fisher’s exact testing did not reveal a significant association between diet group and increased LVIDd (P = 0.06). There were 5 instances of elevated LVIDd measures in the NTD group and 0 instances of elevated LVIDd measures in the TD group with 38 and 43 normal LVIDd measurements respectively resulting in a relative risk of 11 (95% CI 0.63 to 193.0, z statistic 1.64, P = 0.10) (Fig 3D). Diet brand, variety, instances of low taurine events and the identified echocardiographic changes indicative of systolic dysfunction are listed in Tables 3 and 4 for dogs eating a TD and NTD respectively.

**Table 1:** Diet brands, varieties and characteristics for golden retrievers fed a Traditional Diet (TD).

Diet Brand	Diet Variety	T	M	GF	LP	LP in Top 5	No. of LP Ingredients
1	A	N	N	N	N	N	0
	B	N	N	N	N	N	0
	C	N	N	N	N	N	0
	D	N	N	N	Y	N	2
	E	N	N	N	N	N	0
	F	N	N	N	N	N	0
	G	N	N	N	N	N	0
2	A	N	N	N	N	N	0
3	A	N	N	N	N	N	0
	B	N	N	N	N	N	0
4	A	Y	Y	N	N	N	0
	B	Y	Y	N	N	N	0
	C	N	Y	N	N	N	0
	D	Y	N	N	N	N	0
5	A	N	Y	N	N	N	0
	B	N	Y	N	N	N	0

List of group 1 traditional diet brands, their respective varieties, and diet characteristics. For each diet variety we list (Y = yes or N = No) whether T = taurine or M = methionine was added to the diet as well if it was a GF = grain free diet and if it contained LP = legumes or potatoes.

1A = Purina Pro Plan Focus Adult Sensitive Skin and Stomach Salmon and Rice Formula Dry Dog Food

1B = Purina Pro Plan Sport Performance 30/20 Formula Dry Dog Food

1C = Purina Pro Plan Bright Mind Adult Chicken and Rice Formula Dry Dog Food

1D = Purina Pro Plan Bright Mind Adult 7+ Turkey and Rice Formula Dry Dog Food

1E = Purina Pro Plan Savor Shredded Blend Adult Chicken and Rice Formula Dry Dog Food

1F = Purina Pro Plan Focus Puppy Large Breed Chicken and Rice Formula Dry Dog Food

1G = Purina Pro Plan Focus Adult Large Breed Formula Dry Dog Food

2A = Purina One SmartBlend Healthy Weight Formula Adult Premium Dog Food (dry)

3A = Purina Pro Plan Veterinary Diets OM Overweight Management Canine Formula (dry)

3B = Purina Pro Plan Veterinary Diets EN Gastroenteric Canine Formula (dry)

4A = Royal Canin Canine Gastrointestinal Low-Fat Dry Dog Food

4B = Royal Canin Golden Retriever Adult Dry Dog Food

4C = Royal Canin Large Adult Dry Dog Food

4D = Royal Canin Golden Retriever Puppy Dry Dog Food

5A = Eukanuba Adult Large Breed Dog Food (dry)

5B = Eukanuba Performance Dog Food: Active Dog Food (dry)

**Table 2:** Diet brands, varieties and characteristics for golden retrievers fed a non-traditional diet (NTD).

Diet Brand	Diet Variety	T	M	GF	LP	LP in Top 5	No. of LP Ingredients
1	A	N	N	Y	Y	N	7
	B	N	N	Y	Y	N	6
	C	N	N	Y	Y	Y	7
	D	N	N	Y	Y	N	7
2	A	N	N	*	*	*	*
3	A	N	N	Y	Y	Y	1
	B	N	N	Y	Y	Y	1
	C	N	N	Y	Y	Y	1
	D	N	N	Y	Y	Y	1
	E	N	N	Y	Y	Y	2
4	A	N	N	Y	Y	Y	1
	B	N	N	Y	Y	Y	1
5	A	Y	N	Y	Y	Y	5
	B	N	N	Y	Y	Y	4
	C	N	N	Y	Y	Y	5
	D	Y	Y	Y	Y	Y	5
6	A	Y	Y	N	Y	N	3
	B	Y	Y	N	Y	N	2
	C	Y	N	Y	Y	N	6
	D	Y	N	Y	Y	Y	4
7	A	N	N	Y	Y	Y	1
	B	N	N	Y	Y	Y	1
	C	N	N	Y	Y	Y	1
8	A	N	N	N	Y	Y	1
	B	Y	Y	Y	Y	Y	1
	C	N	Y	N	Y	Y	4
9	A	N	N	Y	Y	Y	1
10	A	Y	N	Y	N	N	0
11	A	N	N	Y	N	N	0
	B	N	N	Y	N	N	0
12	A	Y	N	N	N	N	0
13	A	N	N	Y	N	N	0
14	A	N	N	Y	N	N	0
15	A	Y	Y	Y	Y	Y	4
16	A	Y	Y	Y	Y	Y	3
	B	Y	Y	Y	Y	Y	2
	C	N	N	Y	Y	N	2
	D	Y	Y	Y	Y	Y	4
17	A	Y	Y	Y	Y	Y	3
18	A	N	N	Y	Y	N	3
	B	N	N	Y	Y	Y	2
19	A	N	N	Y	Y	Y	5
	B	N	N	Y	Y	Y	6
	C	N	N	Y	Y	Y	3
	D	N	N	N	Y	N	2
	E	N	N	Y	Y	N	1
20	A	N	N	Y	N	N	0
	B	N	N	Y	N	N	0
	C	N	N	Y	N	N	0
	D	N	N	Y	N	N	0
	E	N	N	Y	N	N	0
	F	N	N	Y	N	N	0
21	A	N	N	Y	Y	Y	1
	B	N	N	Y	Y	N	1
22	A	N	N	Y	Y	N	2
23	A	N	N	Y	Y	N	7
	B	N	N	Y	Y	N	8
24	A	Y	Y	Y	Y	N	6
25	A	N	N	Y	N	N	0
26	A	Y	N	Y	Y	Y	5
27	A	Y	Y	Y	Y	Y	4

List of group 2 non-traditional diet brands, their respective varieties, and diet characteristics. For each diet variety we list (Y = yes or N = No) whether T = taurine or M = methionine was added to the diet as well if it was a GF = grain free diet and if it contained LP = legumes or potatoes.

* indicates that information is not available for the given diet.

1A = ACANA Pacifica (dry)

1B = ACANA Meadowland (dry)

1C = ACANA Singles Lamb and Apple (dry)

1D = ACANA Grasslands (dry)

2A = Greentripe Xkaliber: Green Tripe, Heart, Tongue, Trachea and Ground Bone (raw)

3A = The Honest Kitchen Dehydrated–Grain Free Turkey Recipe (Embark)

3B = The Honest Kitchen Dehydrated–Whole Grain Turkey Recipe (Keen)

3C = The Honest Kitchen Dehydrated–Grain Free Fish Recipe (Zeal)

3D = The Honest Kitchen Dehydrated–Limited Ingredient Fish Recipe (Brave)

3E = The Honest Kitchen Dehydrated–Grain Free Fruit and Veggie Base Mix (Preference)

4A = Instinct by Nature’s Variety Original Grain-Free Recipe with Real Duck (dry)

4B = Instinct by Nature’s Variety Limited Ingredient Diet Grain-Free Recipe with Real Turkey (dry)

5A = Taste of the Wild High Prairie Canine Recipe with Roasted Bison and Roasted Venison (dry)

5B = Taste of the Wild Wetlands Canine Recipe with Roasted Fowl (dry)

5C = Taste of the Wild Pacific Stream Canine Recipe with Roasted Salmon (dry)

5D = Taste of the Wild Sierra Mountain Canine Recipe with Roasted Lamb (dry)

6A = Fromm Weight Management Gold (dry)

6B = Fromm Adult Gold (dry)

6C = Fromm Salmon a La Veg Recipe (dry)

6D = Fromm Lamb and Lentil Recipe (dry)

7A = Sport Dog Food Elite Grain Free Chicken Meal 30/14 (dry)

7B = Sport Dog Food Elite Grain Free Whitefish Meal 30/14 (dry)

7C = Sport Dog Food Working Dog–Grain and Peas Free Turkey Formula (dry)

8A = Nutri Source Pure Vita Duck and Oatmeal (dry)

8B = Nutri Source Pure Vita Salmon Entrée (canned)

8C = Nutri Source Weight Management Dog Food Chicken and Chicken Meal Protein (dry)

9A = Sojos Complete Turkey Recipe (raw)

10A = Stella and Chewy’s Freeze Dried Dinner Patties, Lamb and Venison Flavors (raw)

11A = Oma’s Pride Beef and Veggies (raw)

11B = Oma’s Pride Turkey and Veggies (raw)

12A = Annamaet Ultra Chicken Meal and Brown Rice (dry)

13A = Darwin’s Natural Selections Beef, Turkey and Pork (raw)

14A = Raw Bistro Dog Fare Grass-Fed Beef Entrée

15A = Earthborn Holistic Coastal Catch (dry)

16A = Natural Balance L.I.D. Limited Ingredient Diets Grain Free Potato and Duck Dry Dog Food Formula

16B = Natural Balance L.I.D. Limited Ingredient Diets Grain Free Sweet Potato and Fish Dry Dog Food Formula

16C = Natural Balance L.I.D. Limited Ingredient Diets Grain Free Chicken and Sweet Potato Canned Dog Formula

16D = Natural Balance L.I.D. Limited Ingredient Diets Grain Free Bison and Sweet Potato Dry Dog Food Formula

17A = Victor Grain Free Yukon River Canine (dry)

18A = Merrick Grain Free Wilderness Blend in Gravy (canned)

18B = Merrick Chunky Grain Free Big Texas Steak Tips Dinner in Gravy (canned)

19A = Kirkland Signature Nature’s Domain Salmon Meal and Sweet Potato Dog Food (dry)

19B = Kirkland Signature Nature’s Domain Beef Meal and Sweet Potato Dog Food (dry)

19C = Kirkland Signature Nature’s Domain Turkey Meal and Sweet Potato Dog Food (dry)

19D = Kirkland Signature Adult Formula Chicken, Rice and Vegetable Dog Food (dry)

19E = Kirkland Signature Nature’s Domain Turkey and Pea Stew for Dogs (canned)

20A = Top Quality Dog Food Beef HVM (raw)

20B = Top Quality Dog Food Chicken HVM (raw)

20C = Top Quality Dog Food Pork HVM (raw)

20D = Top Quality Dog Food Green Tripe Ground (raw)

20E = Top Quality Dog Food Green Tripe Chunks (raw)

20F = Top Quality Dog Food Beef with Tripe and Organ Meats (raw)

21A = K-9 Kravings Beef and Vegetables (raw)

21B = K-9 Kravings Chicken, Beef and Vegetables (raw)

22A = Canidae Grain Free Pure Ancestral Fish Formula Raw Coated Dry Dog Food

23A = Orijen Original Dog Food (dry)

23B = Orijen Tundra Dog Food (dry)

24A = Blue Buffalo Life Protection Formula Lamb and Brown Rice Recipe (dry)

25A = Primal Freeze-Dried Nuggets, Beef and Duck Flavor (raw)

26A = Wellness Core Grain Free Large Breed (dry)

27A = Zignature Lamb Formula (dry)

**Table 3:** Diet brands, variety, instances of low taurine events and echocardiographic changes indicative of systolic dysfunction for golden retrievers being fed a traditional diet.

Diet Brand	Diet Variety	No. of dogs	No. of low taurine events	No. of dogs with high LVIDd	No. of dogs with high LVIDs	No. of dogs with Low FS
1	A	11	0	0	0	0
	B	8	0	0	0	0
	C	1	0	0	0	0
	D	2	0	0	1	0
	E	4	0	0	0	0
	F	1	0	0	0	0
	G	4	0	0	0	0
2	A	1	0	0	0	0
3	A	1	0	0	0	0
	B	1	0	0	0	0
4	A	1	0	0	0	0
	B	6	0	0	0	0
	C	3	0	0	0	0
	D	1	0	0	0	0
5	A	1	1	0	0	0
	B	2	0	0	0	0

List of group 1 traditional diet brands and their respective varieties (Listed in Table 1). For each diet variety we report the number of dogs with low taurine events (plasma and whole blood) and echocardiographic changes indicative of systolic dysfunction. Note some dogs were being fed multiple diets, so the number of dogs in this table does not equal the number of dogs enrolled in this study.

**Table 4:** Diet brands, variety, instances of low taurine events and echocardiographic changes indicative of systolic dysfunction for golden retrievers being fed a non-traditional diet.

Diet Brand	Diet Variety	No. of dogs	No. of low taurine events	No. of dogs with high LVIDd	No. of dogs with high LVIDs	No. of dogs with Low FS
1	A	1	0	0	0	0
	B	1	0	0	0	0
	C	1	2	1	1	1
	D	1	0	1	1	0
2	A	1	1	0	0	0
3	A	3	2	0	0	0
	B	2	0	0	0	0
	C	1	0	0	0	0
	D	1	0	0	0	0
	E	1	0	0	0	0
4	A	1	0	0	0	0
	B	1	0	0	0	0
5	A	1	0	0	0	0
	B	1	0	0	0	0
	C	1	0	0	0	0
	D	2	1	0	2	2
6	A	1	1	0	0	0
	B	2	0	0	0	0
	C	1	0	0	0	0
	D	2	1	0	2	2
7	A	1	0	0	0	0
	B	1	0	0	0	0
	C	1	0	0	0	0
8	A	1	0	0	0	0
	B	1	0	0	0	0
	C	1	1	0	0	1
9	A	1	0	0	0	0
10	A	1	0	0	0	0
11	A	1	0	0	0	0
	B	1	0	0	0	0
12	A	1	0	0	0	0
13	A	1	0	0	0	0
14	A	1	0	0	0	0
15	A	1	0	0	0	0
16	A	3	0	0	0	0
	B	2	0	1	1	0
	C	2	0	1	1	0
	D	1	0	0	0	0
17	A	2	0	0	0	0
18	A	2	0	0	0	0
	B	2	0	0	0	0
19	A	3	0	2	2	1
	B	1	0	0	0	0
	C	1	0	0	0	0
	D	1	0	0	0	0
	E	1	0	0	0	0
20	A	2	1	0	0	0
	B	2	1	0	0	0
	C	2	1	0	0	0
	D	2	1	0	0	0
	E	2	1	0	0	0
	F	2	1	0	0	0
21	A	2	1	0	0	0
	B	2	1	0	0	0
22	A	1	0	0	0	0
23	A	2	0	0	0	1
	B	1	0	0	0	0
24	A	1	0	0	0	1
25	A	1	0	0	0	1
26	A	1	0	0	1	0
27	A	1	2	0	1	1

List of group 2 non-traditional diet brands and their respective varieties (Listed in Table 2). For each diet variety we report the number of dogs with low taurine events (plasma and whole blood) and echocardiographic changes indicative of systolic dysfunction. Note some dogs were being fed multiple diets, so the number of dogs in this table does not equal the number of dogs enrolled in this study.

**Fig 4:**
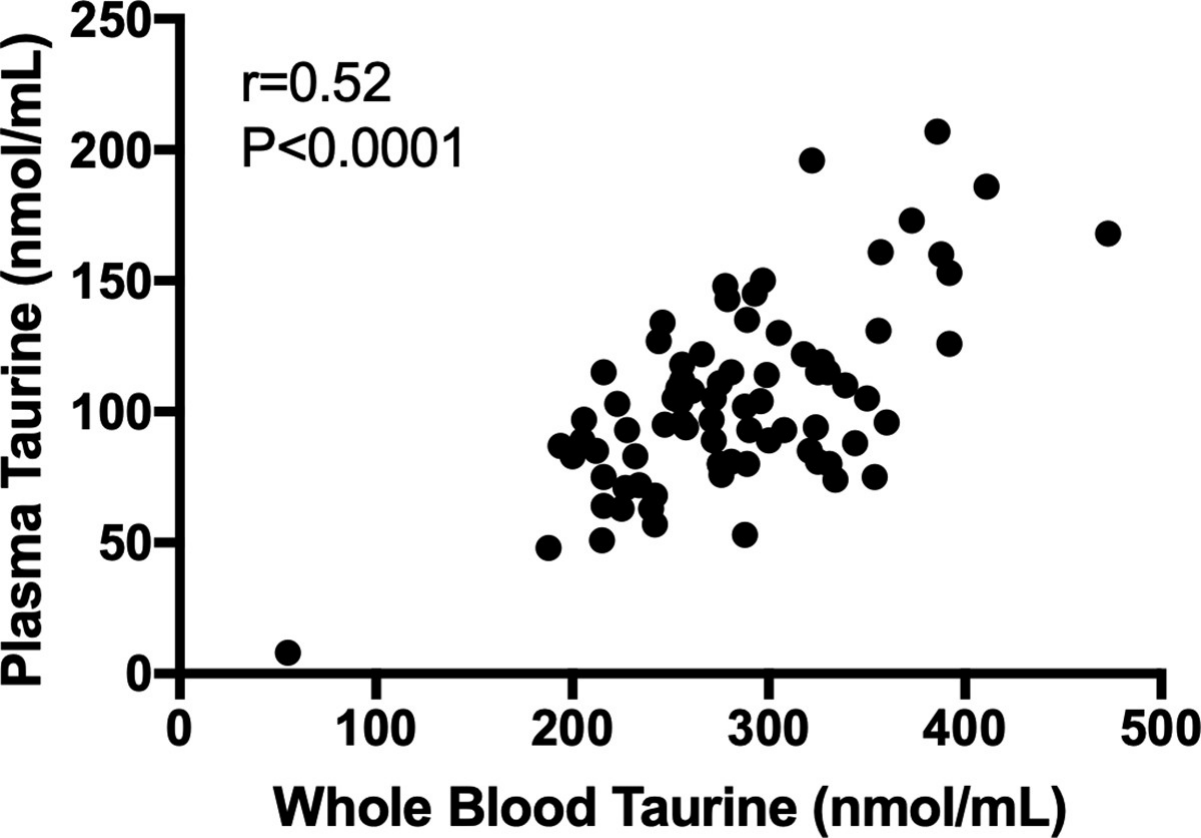
Spearman non-parametric correlation analysis result for whole-blood taurine concentration (nmol/mL) vs. plasma taurine concentration (nmol/mL).

For the 79 dogs where both whole blood and plasma taurine concentrations were available, Spearman correlation evaluation revealed a significant moderate correlation (P<0.0001; r = 0.52, r^2^ = 0.27) (Fig 4).

### Food intake & time on diet

**Fig 5:**
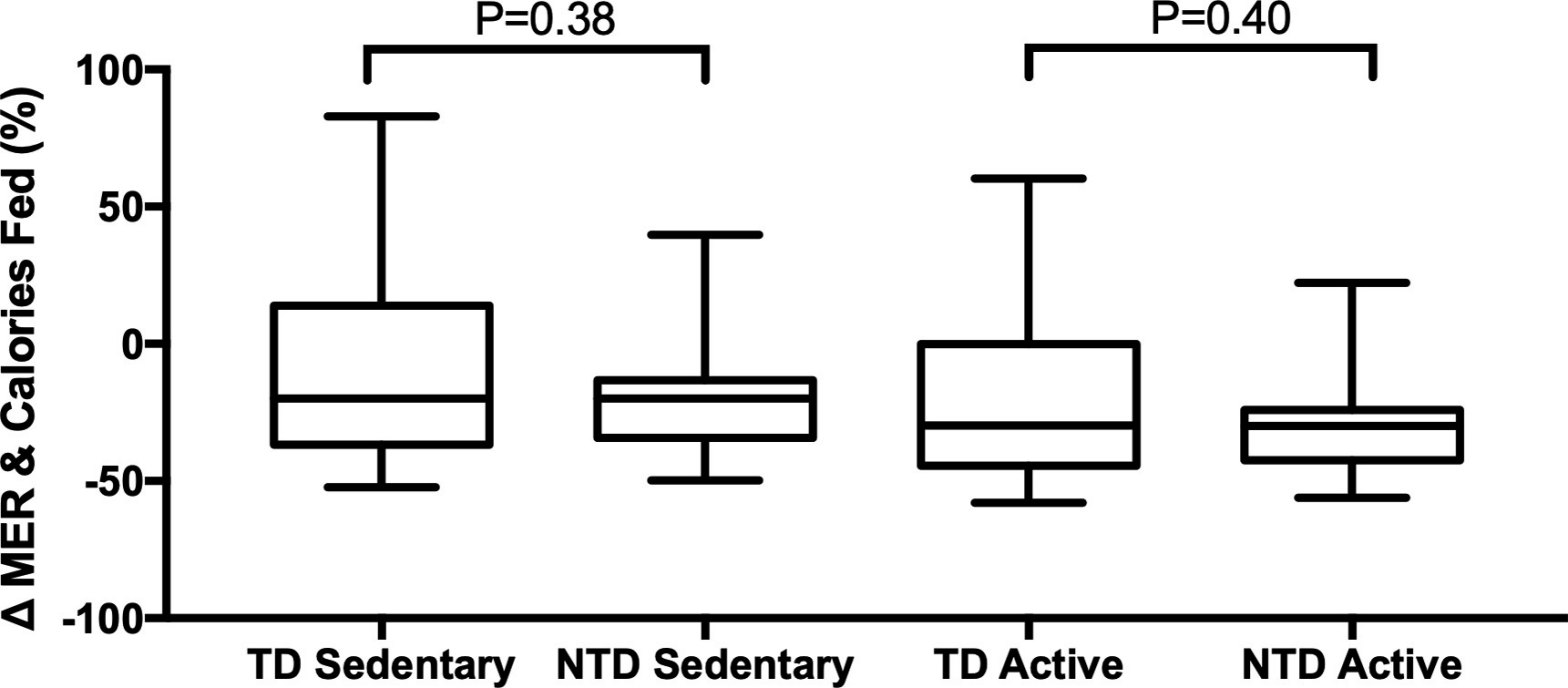
Illustration of the differences in maintenance energy requirement and calories consumed for dogs fed a traditional (TD) and non-traditional (NTD) diet for both a sedentary and active lifestyle.

Dogs in both groups were typically fed fewer calories when compared to sedentary or active MER calculations. The median percent difference between the number of calories fed and the calculated sedentary or active energy requirements was -19.9% (IQR -36.7, 14.0) and -30.0% (IQR -44.6, -0.30) respectively for the TD group. The median percent difference between the amount fed and the calculated sedentary or active energy requirements was -20.0% (IQR -34.2, -13.32) and -30.0% (IQR -42.4, -24.2) respectively for the NTD group. No difference was identified when comparing the TD to the NTD group for either sedentary (P = 0.38) or active (P = 0.38) MER calculations (Fig 5). Diet calculations are shown for the TD and NTD groups in Tables 5 and 6 respectively. The median number of months a dog was fed a diet was shorter in the TD group with a median of 7 (IQR 5.0, 21.0) compared to the NTD group with a median of 18 (IQR 9.0, 36.0; P = 0.006). There were a total of 6 dogs in the TD group and 1 dog in the NTD group that were fed their diet for the minimum of 3 months prior to study enrollment.

**Table 5:** Calculated Resting Energy Requirement (RER) and Maintenance Energy Requirement (MER) range for golden retrievers (n = 43) fed a Traditional Diet (TD) and the difference in actual vs. MER for a sedentary and active lifestyle.

Subject No.	Weight (kg)	BCS	Diet	Calculated RER (kcal/day)	MER Range (kcal/day)	% Difference in actual vs MER	
						Sedentary	Active
1	33.18	5	1A	968	1355–1548	-36.67	-44.59
2	23.64	5	1B	750	1051–1201	-39.86	-47.38
3	40.91	5	1B	1132	1585–1812	19.86	4.87
4^1^	27.73	5	1A	846	1184–1353	9.32	-4.35
			1C				
5	31.82	5	1B	938	1313–1500	8.54	-5.03
6	17.95	4	4A	611	855–977	-43.85	-50.86
7	36.36	5	4B	1037	1451–1659	-42.94	-50.08
8	26.36	5	1A	814	1140–1303	-5.94	-17.70
9	30.23	5	4C	902	1263–1444	-14.51	-25.20
10	24.09	5	1D	761	1066–1218	-20.61	-30.54
11	25.45	5	1D	793	1111–1269	-23.82	-33.35
12	28.80	6	5A	870	1218–1392	-45.66	-52.46
13	25.90	5	4B	804	1125–1286	-50.94	-57.07
14	25.10	5	4B	785	1099–1256	-49.77	-56.05
15	23.70	4.5	2A	752	1053–1203	-39.20	-46.80
16	22.85	5	4C	732	1024–1171	-29.70	-38.49
17	20.55	4	4C	676	946–1081	-23.88	-33.40
18	26.80	5	4B	825	1154–1319	-52.18	-58.16
19	34.00	6	1E	986	1380–1577	10.74	-3.11
20	27.25	5	1E	835	1169–1336	30.73	14.39
21	27.80	6	3A	847	1186–1356	-23.72	-33.26
22	36.10	5	1B	1031	1443–1649	31.64	15.19
23	25.10	5	1E	785	1099–1256	-13.10	-23.96
24	25.75	5	1E	800	1120–1280	-14.75	-25.41
25	25.50	5	1F	794	1112–1271	40.62	23.04
26	32.65	5	1A	956	1339–1530	-19.88	-29.89
27	25.20	4.5	1B	787	1102–1260	-35.36	-43.44
28^2^	31.30	7	1A	926	1297–1482	7.25	-13.30
			1B				
			5B				
29^2^	38.75	7	1A	1087	1522–1739	82.95	60.08
			1B 5B				
30	22.70	5	1G	728	1019–1165	-22.29	-32.00
31	32.75	6	4B	958	1342–1533	-28.00	-37.00
32	29.30	6	4B	882	1234–1410	-10.55	-21.73
33^3^	29.30	6	1G	882	1234–1410	-35.83	-43.85
34	27.30	6	1B	836	1170–1338	21.75	6.53
35	31.70	6	1A	935	1309–1496	14.68	0.35
36	34.30	6	1G	992	1389–1587	14.04	-0.21
37	27.30	7	1A	836	1170–1338	-45.02	-51.89
38	29.50	5	1A	886	1240–1418	-48.13	-54.61
39	30.55	6	1A	910	1273–1455	-32.62	-41.05
40	37.75	7	1A	1066	1492–1706	-13.77	-24.55
41	28.35	5	3B	860	1204–1376	65.69	44.98
42	32.25	5	1G	947	1326–1516	19.44	4.51
43	27.80	5	4D	847	1186–1356	13.95	-0.29

List of individual weight, body condition score (BCS), diet brand and variety, calculated resting energy requirement (RER) in kilocalories per day (kcal/day), calculated maintenance energy requirement (MER) in kilocalories per day (kcal/day), and percent (%) difference in actual amount of diet fed (kcal/day) to calculated MER for group 1 diets. Maintenance energy requirements were listed as a range of RER multiplied by a factor of 1.4–1.6 to account for a less active or more active lifestyle, respectively. For dogs being fed multiple diets, % difference in actual vs MER was calculated as an average between the different diets.

^1^ Calculations for % difference in actual vs MER for subject number 4 were done by averaging the daily kilocalorie recommendations by the manufacturer for both diets.

^2^ Subject numbers 28 and 29 were alternating diets between diets 1A and 1B with diet 5B. Calculations for % difference in actual vs MER were done by averaging the daily kilocalorie recommendations by the manufacturer for all three diets.

^3^ Subject number 33 was occasionally fed diet 1B, but calculations were done based on the primary diet being fed.

1A = Purina Pro Plan Focus Adult Sensitive Skin and Stomach Salmon and Rice Formula Dry Dog Food

1B = Purina Pro Plan Sport Performance 30/20 Formula Dry Dog Food

1C = Purina Pro Plan Bright Mind Adult Chicken and Rice Formula Dry Dog Food

1D = Purina Pro Plan Bright Mind Adult 7+ Turkey and Rice Formula Dry Dog Food

1E = Purina Pro Plan Savor Shredded Blend Adult Chicken and Rice Formula Dry Dog Food

1F = Purina Pro Plan Focus Puppy Large Breed Chicken and Rice Formula Dry Dog Food

1G = Purina Pro Plan Focus Adult Large Breed Formula Dry Dog Food

2A = Purina One SmartBlend Healthy Weight Formula Adult Premium Dog Food (dry)

3A = Purina Pro Plan Veterinary Diets OM Overweight Management Canine Formula (dry)

3B = Purina Pro Plan Veterinary Diets EN Gastroenteric Canine Formula (dry)

4A = Royal Canin Canine Gastrointestinal Low-Fat Dry Dog Food

4B = Royal Canin Golden Retriever Adult Dry Dog Food

4C = Royal Canin Large Adult Dry Dog Food

4D = Royal Canin Golden Retriever Puppy Dry Dog Food

5A = Eukanuba Adult Large Breed Dog Food (dry)

5B = Eukanuba Performance Dog Food: Active Dog Food (dry)

**Table 6:** Calculated Resting Energy Requirement (RER) and Maintenance Energy Requirement (MER) range for golden retrievers (n = 43) fed a non-Traditional Diet (NTD) and the difference in number of calories fed compared to calculated MER for a sedentary and active lifestyle.

Subject No.	Weight (kg)	BCS	Diet	Calculated RER (kcal/day)	MER Range (kcal/day)	% Difference in actual vs MER	
						Sedentary	Active
1	25.91	5	1A	835	1170–1337	-21.00	-30.87
2	31.59	5	2A	933	1306–1492	*	*
			3A				
3	28.64	5	3A	867	1213–1386	*	*
			3B				
			4A				
			4B				
			5A				
			5B				
4	34.09	5	6A	988	1383–1580	*	*
5	21.36	5	3A	696	974–1113	*	*
			3B				
			7A				
6	22.73	4	8A	729	1020–1166	-13.93	-24.69
			9A				
7	29.09	5	3C	877	1228–1403	*	*
			7B				
			10A				
8	28.59	5	11A	866	1212–1358	-24.90	-34.29
			11B				
9	27.23	4	6B	834	1168–1335	39.71	22.25
10	35.77	5	12A	1024	1433–1638	0.46	-12.10
11	28.18	5	5C	856	1199–1370	*	*
12	26.82	5	13A	825	1155–1320	*	*
13	23.64	4	14A	750	1051–1201	*	*
14	35.45	5	15A	1017	1424–1627	-18.36	-28.57
			16D				
15	30.91	5	1B	918	1285–1468	-38.97	-46.60
16	27.27	4	6C	835	1170–1337	1.83	-10.90
17	33.18	5	7C	968	1355–1548	-25.90	-35.16
18	27.73	4	16A	846	1184–1353	-26.11	-35.34
19	25.00	4	16A	783	1096–1252	-4.17	-16.15
20	35.91	6	17A	1027	1438–1643	-36.59	-44.52
			18A				
			18B				
21	30.45	6	17A	907	1270–1452	-28.25	-37.22
			18A				
			18B				
22	30.00	5	19A	897	1256–1436	-46.51	-53.19
23	32.73	9	20A	958	1341–1532	*	*
			20B				
			20C				
			20D				
			20E				
			20F				
			21A				
			21B				
24	26.36	5	16B	814	1140–1303	*	*
			16C				
25	30.91	5	16B	918	1285–1468	*	*
			16C				
26	31.36	5	20A	928	1299–1484	*	*
			20B				
			20C				
			20D				
			20E				
			20F				
			21A				
			21B				
27	34.09	7	3D	988	1383–1580	*	*
			8B				
			22A				
28	33.64	6	19A	978	1369–1564	*	*
			19B				
			19C				
29	30.30	6	6B	904	1266–1446	-11.47	-22.53
30	35.45	5	23A	1017	1424–1627	-15.80	-26.32
31	34.45	5	16A	995	1394–1593	-49.77	-56.05
32	40.25	7	19C	1119	1566–1790	*	*
			19E				
33	36.00	7	19D	1029	1440–1646	-18.14	-28.37
34	29.20	5	23A	879	1231–1407	-32.82	-41.22
			24A				
			25A				
35	34.09	6	5D	988	1383–1580	-33.46	-41.78
			6D				
36	25.45	5	5D	793	1111–1269	-17.16	-27.52
			6D				
37	36.36	5	1C	1037	1451–1659	-18.96	-29.09
38	40.45	6	26A	1123	1572–1797	*	*
39	28.18	5	3E	856	1199–1370	*	*
			23B				
40	37.73	7	1D	1066	1492–1705	-47.98	-54.49
41	35.00	6	19A	1007	1410–1612	7.22	-6.18
42	25.45	6	8C	793	1111–1269	-36.43	-44.38
43	38.64	7	27A	1085	1519–1736	-14.07	-24.81

List of individual weight, body condition score (BCS), diet brand and variety, calculated resting energy requirement (RER) in kilocalories per day (kcal/day), calculated maintenance energy requirement (MER) in kilocalories per day (kcal/day), and percent (%) difference in actual amount of diet fed (kcal/day) to calculated MER for group 2 diets. Maintenance energy requirements were listed as a range of RER multiplied by a factor of 1.4–1.6 to account for a less active or more active lifestyle, respectively. For dogs being fed multiple diets, % difference in actual vs MER was calculated as an average between the different diets.

* Information not available or dog received most of calories from other food sources

^1^ This value is an underestimate as only diets 23A and 24A were used to calculate % difference in actual vs MER for subject 34.

1A = ACANA Pacifica (dry)

1B = ACANA Meadowland (dry)

1C = ACANA Singles Lamb and Apple (dry)

1D = ACANA Grasslands (dry)

2A = Greentripe Xkaliber: Green Tripe, Heart, Tongue, Trachea and Ground Bone (raw)

3A = The Honest Kitchen Dehydrated–Grain Free Turkey Recipe (Embark)

3B = The Honest Kitchen Dehydrated–Whole Grain Turkey Recipe (Keen)

3C = The Honest Kitchen Dehydrated–Grain Free Fish Recipe (Zeal)

3D = The Honest Kitchen Dehydrated–Limited Ingredient Fish Recipe (Brave)

3E = The Honest Kitchen Dehydrated–Grain Free Fruit and Veggie Base Mix (Preference)

4A = Instinct by Nature’s Variety Original Grain-Free Recipe with Real Duck (dry)

4B = Instinct by Nature’s Variety Limited Ingredient Diet Grain-Free Recipe with Real Turkey (dry)

5A = Taste of the Wild High Prairie Canine Recipe with Roasted Bison and Roasted Venison (dry)

5B = Taste of the Wild Wetlands Canine Recipe with Roasted Fowl (dry)

5C = Taste of the Wild Pacific Stream Canine Recipe with Roasted Salmon (dry)

5D = Taste of the Wild Sierra Mountain Canine Recipe with Roasted Lamb (dry)

6A = Fromm Weight Management Gold (dry)

6B = Fromm Adult Gold (dry)

6C = Fromm Salmon a La Veg Recipe (dry)

6D = Fromm Lamb and Lentil Recipe (dry)

7A = Sport Dog Food Elite Grain Free Chicken Meal 30/14 (dry)

7B = Sport Dog Food Elite Grain Free Whitefish Meal 30/14 (dry)

7C = Sport Dog Food Working Dog–Grain and Peas Free Turkey Formula (dry)

8A = Nutri Source Pure Vita Duck and Oatmeal (dry)

8B = Nutri Source Pure Vita Salmon Entrée (canned)

8C = Nutri Source Weight Management Dog Food Chicken and Chicken Meal Protein (dry)

9A = Sojos Complete Turkey Recipe (raw)

10A = Stella and Chewy’s Freeze Dried Dinner Patties, Lamb and Venison Flavors (raw)

11A = Oma’s Pride Beef and Veggies (raw)

11B = Oma’s Pride Turkey and Veggies (raw)

12A = Annamaet Ultra Chicken Meal and Brown Rice (dry)

13A = Darwin’s Natural Selections Beef, Turkey and Pork (raw)

14A = Raw Bistro Dog Fare Grass-Fed Beef Entrée

15A = Earthborn Holistic Coastal Catch (dry)

16A = Natural Balance L.I.D. Limited Ingredient Diets Grain Free Potato and Duck Dry Dog Food Formula

16B = Natural Balance L.I.D. Limited Ingredient Diets Grain Free Sweet Potato and Fish Dry Dog Food Formula

16C = Natural Balance L.I.D. Limited Ingredient Diets Grain Free Chicken and Sweet Potato Canned Dog Formula

16D = Natural Balance L.I.D. Limited Ingredient Diets Grain Free Bison and Sweet Potato Dry Dog Food Formula

17A = Victor Grain Free Yukon River Canine (dry)

18A = Merrick Grain Free Wilderness Blend in Gravy (canned)

18B = Merrick Chunky Grain Free Big Texas Steak Tips Dinner in Gravy (canned)

19A = Kirkland Signature Nature’s Domain Salmon Meal and Sweet Potato Dog Food (dry)

19B = Kirkland Signature Nature’s Domain Beef Meal and Sweet Potato Dog Food (dry)

19C = Kirkland Signature Nature’s Domain Turkey Meal and Sweet Potato Dog Food (dry)

19D = Kirkland Signature Adult Formula Chicken, Rice and Vegetable Dog Food (dry)

19E = Kirkland Signature Nature’s Domain Turkey and Pea Stew for Dogs (canned)

20A = Top Quality Dog Food Beef HVM (raw)

20B = Top Quality Dog Food Chicken HVM (raw)

20C = Top Quality Dog Food Pork HVM (raw)

20D = Top Quality Dog Food Green Tripe Ground (raw)

20E = Top Quality Dog Food Green Tripe Chunks (raw)

20F = Top Quality Dog Food Beef with Tripe and Organ Meats (raw)

21A = K-9 Kravings Beef and Vegetables (raw)

21B = K-9 Kravings Chicken, Beef and Vegetables (raw)

22A = Canidae Grain Free Pure Ancestral Fish Formula Raw Coated Dry Dog Food

23A = Orijen Original Dog Food (dry)

23B = Orijen Tundra Dog Food (dry)

24A = Blue Buffalo Life Protection Formula Lamb and Brown Rice Recipe (dry)

25A = Primal Freeze-Dried Nuggets, Beef and Duck Flavor (raw)

26A = Wellness Core Grain Free Large Breed (dry)

27A = Zignature Lamb Formula (dry)

### Whole blood and plasma taurine reference intervals

**Fig 6:**
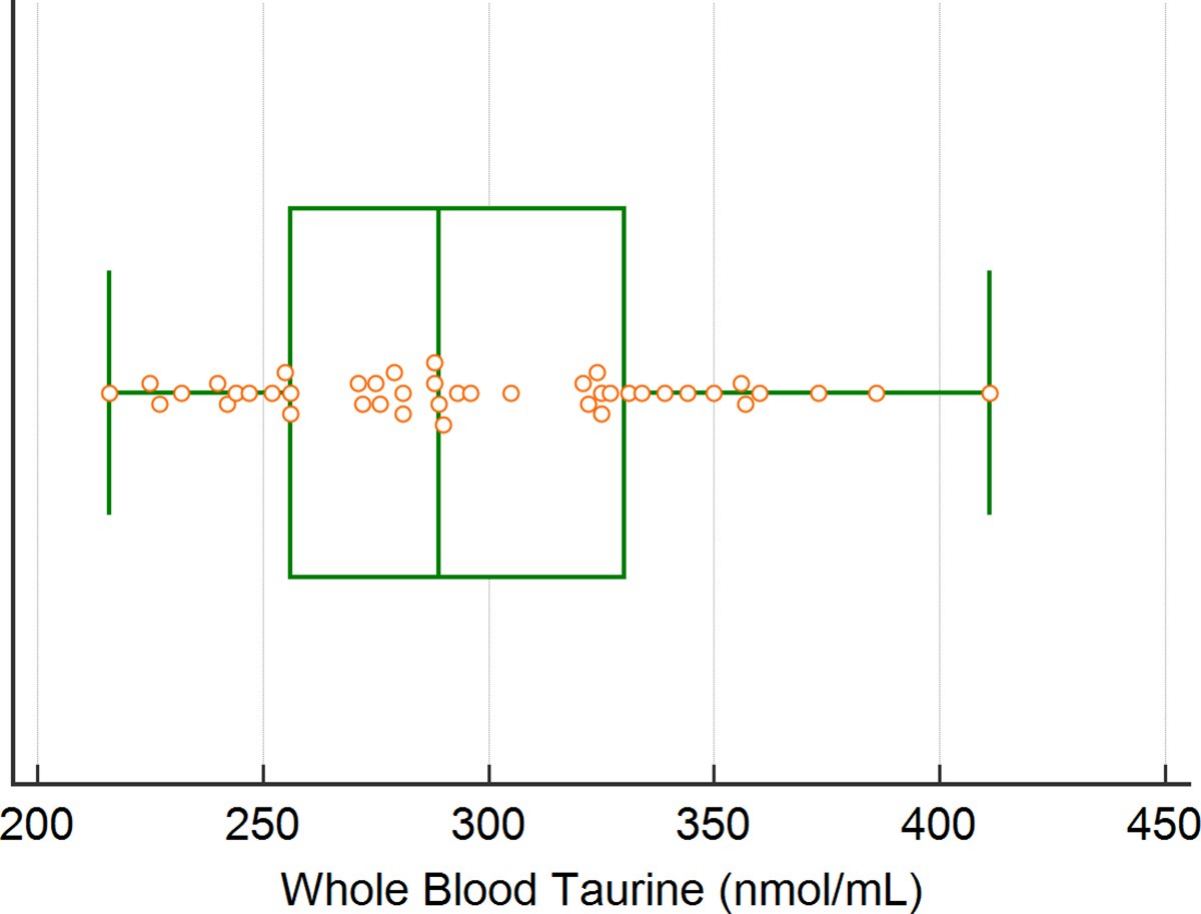
Illustration of the reference interval for whole-blood taurine concentration (nmol/mL).

**Fig 7:**
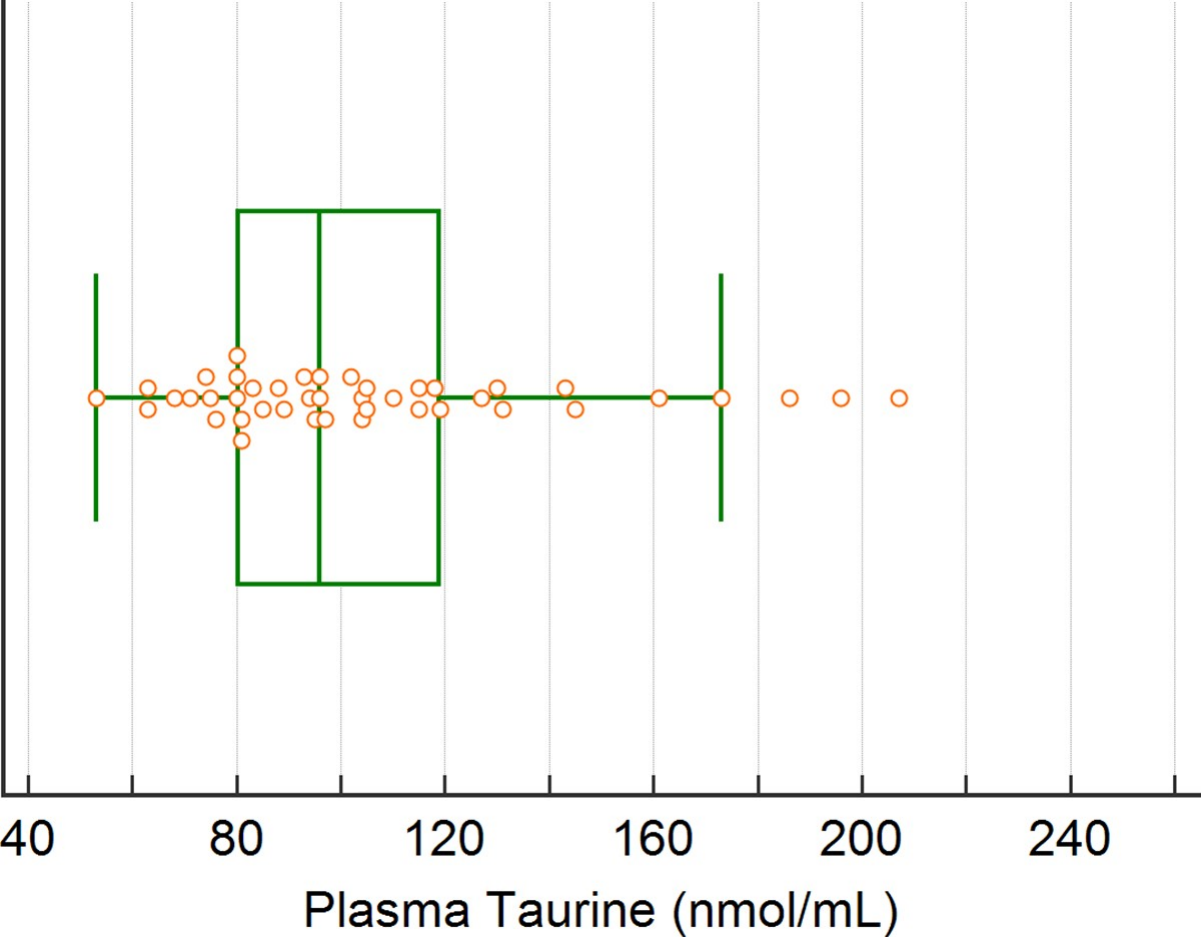
Illustration of the reference interval for plasma taurine concentration (nmol/mL).

Whole blood and plasma taurine reference ranges were generated using the TD diet group which included 43 dogs with normal echocardiographic examinations. For whole blood the robust methodology with 90% bootstrap confidence intervals resulted in a reference interval of 213 to 377 nmol/mL (90% CI lower limit 198 to 230 and upper limit 355 to 396) (Fig 6). For plasma taurine concentration the nonparametric percentile method resulted in a reference interval of 63 to 194 nmol/mL (Fig 7).

## Discussion

Although the dietary link between low taurine concentration and dilated cardiomyopathy has been described and understood for many years, a recent FDA alert identified an emerging nutritionally-mediated cardiomyopathy in dogs [31,32]. Of the dogs reported by the FDA, the golden retriever is the most over-represented [32]. Additionally, a clinical case series of golden retrievers with nutritionally-mediated dilated cardiomyopathy was published that confirmed the dietary features that the FDA noted to be most frequently observed in affected dogs [29]. Until now, an association between grain free diets containing legumes had not been identified as previous reports lacked the necessary control group or were retrospective in nature [28,29].

In this study we identified that golden retrievers eating diets with certain characteristics (NTD group) are at a higher risk of having low taurine concentrations and/or echocardiographic abnormalities supportive of dilated cardiomyopathy when compared to a control group eating TD. The characteristics of the NTD group were specifically chosen based upon identified diet features that are associated with recent reports of cases of nutritionally-mediated dilated cardiomyopathy [28,29,31,32]. These features include being labeled as grain free, containing pulse ingredients like legumes (peas, lentils, etc.) or potatoes, and being produced by a small company as market share appears inversely proportional to the number of reported cases in the FDA consumer complaint dataset. The control group (TD group) was selected to avoid these features and required the company producing the product to be large as previously defined [35], not include pulse ingredients within the top 5 dietary ingredients, be grain inclusive and be a dry kibble diet.

This study also identified that regardless of diet group, golden retrievers are on average fed fewer calories relative to the commonly accepted MER for sedentary or active dogs. This finding was observed in a previous publication and was hypothesized to possibly play a role in the development of nutritionally-mediated DCM [29]. The data in the current study argues that being fed below MER is not a significant factor in the development of low taurine concentrations or echocardiographic abnormalities as feeding below MER was no different between the TD and NTD groups.

Golden retrievers have been previously reported to have taurine-deficient dilated cardiomyopathy and it has been speculated that they may be a more sensitive breed for developing this condition [20,22,23,29]. However, the golden retriever is not a breed with a demonstrated heritable form of dilated cardiomyopathy and until now the number of cases of golden retrievers with DCM in the literature is low, particularly given the breeds extreme popularity [1,2,52–55]. These features make a genetic form of dilated cardiomyopathy within the golden retriever breed exceedingly unlikely. The implication that they may be more sensitive as a breed to taurine deficiency prompted the authors to use the control group (TD group) of this study to construct reference intervals. The reference intervals identified through this study show that the golden retriever may have a slightly higher taurine concentration in health when compared to previous multi-breed studies and the currently adopted reference intervals from the testing laboratory [45,46]. Although the number of golden retrievers used for this reference interval was moderate (n = 43) it is possible that a more robust reference interval cohort may alter the results and as such this recommendation should be critically evaluated. It is also important to observe that in this study the whole blood taurine and not plasma taurine concentrations were significantly lower in the NTD group when compared to the TD group. This discrepancy could be secondary to the sensitivity for erroneously elevated plasma taurine concentrations when hemolysis or other microscopic cellular contamination is observed. Hemolysis is not the only change that can impact plasma taurine concentrations. Platelets present the highest taurine concentrations among cells due to three different types of transporters with varying affinities [56]. Taurine is also the most abundant single amino acid in leukocytes [57]. Taken together, falsely elevated plasma taurine concentrations can arise from any of the above-mentioned cells [56–58]. Although gross hemolysis was not observed in any of the plasma samples in this study, the impact of sample handling and plasma contamination with cells must be considered.

This clinical study has a number of limitations that should be considered. The first is that the authors only analyzed the owner-reported diet history and the dog’s biochemical and echocardiographic data. Thyroid function was not evaluated in this study. Hypothyroidism may influence food intake, body weight and muscle condition, but this variable was not evaluated in this study. Hypothyroidism has also long been considered a variable that may contribute to reduced systolic function [59]. However, recent studies confirm that the key variables of systolic function and chamber dilation measured in this study (FS, LVIDd and LVIDs) were not significantly different between dogs with untreated hypothyroidism and control dogs [59]. Significant hypothyroidism is not expected in any of the study participants, as they were free of clinical signs of this condition. Additionally, more rigorous screening tests were not performed as they were determined to be beyond the scope and budget of this study. While the yield of these diagnostic tests in patients without clinical signs is expected to be low, future studies may consider parasite testing, biochemical profiles, cardiac biomarkers, infectious disease testing, ambulatory electrocardiography monitoring and complete blood counts as part of the health screening at time of enrollment. These additional screening tests may be helpful in ruling out rare conditions that may contribute to reduced systolic function. No diet samples were collected or tested in this study. While analysis of the sulfur amino acid content of the diets may have yielded some insights, quantitative analysis of methionine and cysteine does not provide information about the digestibility and bioavailability of these amino acids in the diet. Evaluating the diets by conducting digestibility and bioavailability studies, and comparing findings to patient data may represent future directions for evaluating this condition. Additionally, the diet ingredient data and evaluation of diet characteristics were collected upon completion of the study from Internet archives [41]. When available, the Internet archive closest to the date of examination was used, however, many of the diets did not have archived pages for evaluation. This leads to the possibility that some foods formulations may have changed during the study period, perhaps even in response to previous publications of FDA alerts [28,29,31,32]. The diet histories of the NTD group in particular are highly variable and reflect current feeding practices in healthy golden retrievers. Many dogs were being fed a mix of multiple diets, which limited the authors’ ability to make energy calculations and derive data that could be determined as the result of specific diets. Instead, the authors evaluated the TD and NTD group as a whole and have summarized features of this diet group rather than associated results to individual diets. Certainly, the findings of this study cannot be applied to every diet meeting these dietary characteristics and future controlled diet trial studies may offer additional insight into disease causation. The requirements for study enrollment mandated that dogs be fed a consistent diet for at least three months, which the vast majority of dogs greatly exceeded. However, there were seven dogs that were fed their diet for the minimum enrollment criteria of 3 months. It is possible that previous diet history may have impacted echocardiographic results and these previous diet histories were not obtained or analyzed as part of this study. The time it takes for diet change to induce or reverse changes in systolic function are not known in the dog, particularly in light of this currently observed nutritionally-mediated cardiomyopathy. This represents a limitation of the study that could be avoided in future long-term studies or through continued follow-up examinations. The chosen three month stable diet requirement is however, more than adequate to observe taurine level depletion and repletion based upon a previous study where plasma taurine concentration changes were observed after as little as 5 weeks[45]. Finally, this study provided only a single evaluation of these patients and did not evaluate disease progression or outcome. Future studies evaluating response to grain free diets produced by the larger manufacturers and diet change in a controlled environment would be a valuable addition to the literature as we strive to understand this emerging issue of nutritionally-mediated DCM.

The current study affirmed our hypothesis and further validates the findings of multiple previous studies and the FDA alert [28–32]. Grain free diets, produced by small companies, including legumes within the top 5 ingredients represent a risk for the development of taurine deficiency and echocardiographic abnormalities consistent with DCM in the golden retriever. Golden retriever specific reference intervals for whole blood and plasma taurine concentrations are proposed. Further research to evaluate these findings in additional dog breeds and elucidate the causative mechanisms behind these results is indicated.

## Supporting information

S1 Fig Results for echocardiographic variable differences for traditional and non-traditional diet groups. A) Unpaired t-test results for ejection fraction percent for different diet groups. B) Mann-Whitney test results for end-systolic volume index for different diet groups. C) Mann-Whitney test results for end-diastolic volume index for different diet groups.
(TIF)
