# Expression of Concern: Development of plasma and whole blood taurine reference ranges and identification of dietary features associated with taurine deficiency and dilated cardiomyopathy in golden retrievers: A prospective, observational study

**DOI:** 10.1371/journal.pone.0239078

**Published:** 2020-09-09

**Authors:** The PLOS ONE Editors

After this article [1] was published, concerns were raised about undeclared competing interests, and about the study design and conclusions.

In response to the concerns about declaration of potential competing interests, the authors have provided the following update to the Competing Interests statement:

AJF is the Scientific Director and JY is the Technical Director of the Amino Acid Laboratory at the University of California, Davis (UCD) that provides amino acid analysis on a fee for service basis. AJF received remuneration for lectures or as an advisor on behalf of Nestlé Purina PetCare, Mars Petcare, Synergy Food Ingredients, the Mark Morris and Pet Food Institutes. AJF received funding from Nutro for graduate student training. A resident on the Nutrition Service, mentored in part by AJF, received funds from the Hill’s Pet Nutrition Resident Clinical Study Grants program, matched by the Center for Companion Animal Health, School of Veterinary Medicine at UCD. The Veterinary Medical Teaching Hospital at UCD received funding from Royal Canin to support a resident, and from Nestlé Purina PetCare to partially support a nutrition technician. AJF has a contract with the FDA on unrelated research. Since the time of article submission, JAS has received research support from Nature’s Variety Inc. Collectively, this did not influence the collection or interpretation of results in this study. There are no patents, products in development or marketed products to declare. This does not alter our adherence to PLOS ONE policies on sharing data and materials.

Concerns raised about the study are outlined below. The authors noted that some of these issues were discussed during pre-publication peer review and/or in the published article.

- Questions were raised around the criteria used to categorize diets, including about the rationale of including global company sales details in these criteria given the objectives of the study.
- Concerns were raised about potential confounds in the study, for example due to the inclusion of adult, puppy, and prescription-only foods, and due to inclusion of raw and kibble diets in the non-traditional group whereas the traditional group only included kibble.

- Soybeans are in the legume family, but soybeans and soy products were not considered as legumes for the purpose of categorizing diets in this study. The authors have requested a correction to clarify that this was an intentional exclusion, and they have noted that this aspect of the study design aligned with how soy products are being handled in a related FDA investigation.
- Questions were raised about the statistical analyses reported in the article.
- Reporting errors were noted, including errors in Tables 1 and 2, for which the authors requested a correction, and other issues raised to the journal that may impact categorization of some included foods as traditional versus non-traditional.
- Questions were raised as to whether three months on a given diet is a sufficient duration to impact cardiac/DCM outcomes, and whether diet history prior to a subject’s current diet may impact the study outcomes. Concerns were also raised about differences between groups in the average time on the current diet.

- Questions were raised about the validity of the conclusion statement, “Grain free diets, produced by small companies, including legumes within the top 5 ingredients represent a risk for the development of taurine deficiency and echocardiographic abnormalities consistent with DCM in the golden retriever.”

The article [1] is being reassessed in light of issues raised post-publication, and the journal is investigating the validity of the concerns raised as well as their implications for the reliability of the article’s conclusions. Meanwhile, the *PLOS ONE* Editors issue this Expression of Concern.
