# Correction: Development of plasma and whole blood taurine reference ranges and identification of dietary features associated with taurine deficiency and dilated cardiomyopathy in golden retrievers: a prospective, observational study

**DOI:** 10.1371/journal.pone.0291101

**Published:** 2023-08-31

**Authors:** The PLOS ONE Editors

After this article [1] was published, several concerns were raised as explained in the linked Expression of Concern [2]. In response, *PLOS ONE* reassessed the article with input from an Editorial Board member and a statistical reviewer.

The specific concerns and author responses are outlined below. *PLOS ONE* concluded that with these updates, most issues raised in the Expression of Concern [2] have been resolved, although the authors and consulted experts disagreed about study design concerns discussed below in points 1 and 4.

1. There is substantial variability among individuals in the control group, and there is also heterogeneity between groups that is not adequately addressed in the study design and/or statistical analyses. For example, the article reports between-group differences in the types of food included (raw/dry), body weight, and median time on diet, and within- and between-group differences in the dietary components. The Academic Editor and statistical reviewer both advised that heterogeneity between groups is a substantial concern with the study design that cannot be adequately addressed with a statement regarding study limitations.
  In light of the within- and between-group heterogeneity and the limited statistical analyses that were reported, the conclusions about differences between the groups are not well-supported. For example, conclusions linking diet to risk of echocardiographic abnormalities and dilated cardiomyopathy are not adequately supported.
  In response, the authors noted that while there was substantial variability in diet and general patient characteristics, there was not any great disparity between groups in values that are traditionally associated with DCM (e.g. poor health, age, breed). The variability reflects a limitation of the study design as an observational study rather than a prospective interventional study.
  The authors agreed that, in light of the study design, the conclusions should not declare causative links. The conclusion is updated to:
  Non-traditional diets—defined as grain-free, including non-soy legumes or potatoes, or being produced by a small company—were found in this study to be significantly associated with and have increased relative risk for the identification of echocardiographic abnormalities consistent with nutritionally-mediated DCM. These findings were identifiable in the absence of clinical signs and support the findings of multiple previous studies and ongoing investigations. Additional studies are required to investigate a causative link between the non-traditional diets as defined in this study and the development of nutritionally-mediated DCM.
2. In response to the concern discussed in [2] about using global company sales in categorizing diets, the authors commented that the distinction between large and small pet food companies based on global sales is an accepted methodology in prior published research and was referenced in the Methods (references 35 and 36 in [1]).
3. Some diet details were incorrectly reported in [1].

The authors noted that the following information should be added to the Materials & Methods section:

For the purposes of this study, legume ingredients were counted as pea or pea derivatives, chickpeas, lentils or lentil derivatives, and other beans or bean products (excluding soy). Soy was not classified as a legume for the purpose of this study to align with how soy was classified in a related FDA investigation [3].

Errors in Table 1 and Table 2 are corrected here:

**Table 1:** Diet brands, varieties and characteristics for golden retrievers fed a traditional diet (TD).

Diet Brand	Diet Variety	T	M	GF	LP	LP in Top 5	No. of LP Ingredients
1	A	N	N	N	N	N	0
	B	N	N	N	N	N	0
	C	N	N	N	N	N	0
	D	N	N	N	Y	N	2
	E	N	N	N	N	N	0
	F	N	N	N	Y	N	1
	G	N	N	N	N	N	0
2	A	N	N	N	N	N	0
3	A	N	N	N	Y	N	1
	B	N	N	N	N	N	0
4	A	Y	Y	N	N	N	0
	B	Y	Y	N	N	N	0
	C	N	Y	N	N	N	0
	D	Y	N	N	N	N	0
5	A	N	Y	N	N	N	0
	B	N	Y	N	N	N	0

List of group 1 traditional diet brands, their respective varieties, and diet characteristics. For each diet variety we list (Y = yes or N = No) whether T = taurine or M = methionine was added to the diet as well if it was a GF = grain free diet and if it contained LP = legumes or potatoes.

1A = Purina Pro Plan Focus Adult Sensitive Skin and Stomach Salmon and Rice Formula Dry Dog Food

1B = Purina Pro Plan Sport Performance 30/20 Formula Dry Dog Food

1C = Purina Pro Plan Bright Mind Adult Chicken and Rice Formula Dry Dog Food

1D = Purina Pro Plan Bright Mind Adult 7+ Turkey and Rice Formula Dry Dog Food

1E = Purina Pro Plan Savor Shredded Blend Adult Chicken and Rice Formula Dry Dog Food

1F = Purina Pro Plan Focus Puppy Large Breed Chicken and Rice Formula Dry Dog Food

1G = Purina Pro Plan Focus Adult Large Breed Formula Dry Dog Food

2A = Purina One SmartBlend Healthy Weight Formula Adult Premium Dog Food (dry)

3A = Purina Pro Plan Veterinary Diets OM Overweight Management Canine Formula (dry)

3B = Purina Pro Plan Veterinary Diets EN Gastroenteric Canine Formula (dry)

4A = Royal Canin Canine Gastrointestinal Low-Fat Dry Dog Food

4B = Royal Canin Golden Retriever Adult Dry Dog Food

4C = Royal Canin Large Adult Dry Dog Food

4D = Royal Canin Golden Retriever Puppy Dry Dog Food

5A = Eukanuba Adult Large Breed Dog Food (dry)

5B = Eukanuba Performance Dog Food: Active Dog Food (dry)

**Table 2:** Diet brands, varieties and characteristics for golden retrievers fed a non-traditional diet (NTD).

Diet Brand	Diet Variety	T	M	GF	LP	LP in Top 5	No. of LP Ingredients
1	A	N	N	Y	Y	N	7
	B	N	N	Y	Y	N	6
	C	N	N	Y	Y	Y	7
	D	N	N	Y	Y	N	7
2	A	N	N	*	*	*	*
3	A	N	N	Y	Y	Y	1
	B	N	N	Y	Y	Y	1
	C	N	N	Y	Y	Y	1
	D	N	N	Y	Y	Y	1
	E	N	N	Y	Y	Y	2
4	A	N	N	Y	Y	Y	1
	B	N	N	Y	Y	Y	1
5	A	Y	N	Y	Y	Y	5
	B	N	N	Y	Y	Y	4
	C	N	N	Y	Y	Y	5
	D	Y	Y	Y	Y	Y	5
6	A	Y	Y	N	Y	N	3
	B	Y	Y	N	Y	N	2
	C	Y	N	Y	Y	Y	4
	D	Y	N	Y	Y	Y	4
7	A	N	N	Y	Y	Y	1
	B	N	N	Y	Y	Y	1
	C	N	N	Y	Y	Y	1
8	A	N	N	N	Y	Y	1
	B	Y	Y	Y	Y	Y	1
	C	N	Y	N	Y	Y	4
9	A	N	N	Y	Y	Y	1
10	A	Y	N	Y	N	N	0
11	A	N	N	Y	N	N	0
	B	N	N	Y	N	Y	1
12	A	Y	N	N	N	N	0
13	A	N	N	Y	N	N	0
14	A	N	N	Y	N	N	0
15	A	Y	Y	Y	Y	Y	4
16	A	Y	Y	Y	Y	Y	3
	B	Y	Y	Y	Y	Y	3
	C	N	N	Y	Y	Y	3
	D	Y	Y	Y	Y	Y	4
17	A	Y	Y	Y	Y	Y	3
18	A	N	N	Y	Y	N	3
	B	N	N	Y	Y	Y	2
19	A	N	N	Y	Y	Y	5
	B	N	N	Y	Y	Y	6
	C	N	N	Y	Y	Y	3
	D	N	N	N	Y	N	2
	E	N	N	Y	Y	N	1
20	A	N	N	Y	N	N	0
	B	N	N	Y	N	N	0
	C	N	N	Y	N	N	0
	D	N	N	Y	N	N	0
	E	N	N	Y	N	N	0
	F	N	N	Y	N	N	0
21	A	N	N	Y	Y	Y	1
	B	N	N	Y	Y	N	1
22	A	N	N	Y	Y	N	2
23	A	N	N	Y	Y	N	7
	B	N	N	Y	Y	N	8
24	A	Y	Y	N	Y	N	6
25	A	N	N	Y	N	N	0
26	A	Y	N	Y	Y	Y	5
27	A	Y	Y	Y	Y	Y	4

List of group 2 non-traditional diet brands, their respective varieties, and diet characteristics. For each diet variety we list (Y = yes or N = No) whether T = taurine or M = methionine was added to the diet as well if it was a GF = grain free diet and if it contained LP = legumes or potatoes. * indicates that information is not available for the given diet.

1A = ACANA Pacifica (dry)

1B = ACANA Meadowland (dry)

1C = ACANA Singles Lamb and Apple (dry)

1D = ACANA Grasslands (dry)

2A = Greentripe Xkaliber: Green Tripe, Heart, Tongue, Trachea and Ground Bone (raw)

3A = The Honest Kitchen Dehydrated–Grain Free Turkey Recipe (Embark)

3B = The Honest Kitchen Dehydrated–Whole Grain Turkey Recipe (Keen)

3C = The Honest Kitchen Dehydrated–Grain Free Fish Recipe (Zeal)

3D = The Honest Kitchen Dehydrated–Limited Ingredient Fish Recipe (Brave)

3E = The Honest Kitchen Dehydrated–Grain Free Fruit and Veggie Base Mix (Preference)

4A = Instinct by Nature’s Variety Original Grain-Free Recipe with Real Duck (dry)

4B = Instinct by Nature’s Variety Limited Ingredient Diet Grain-Free Recipe with Real Turkey (dry)

5A = Taste of the Wild High Prairie Canine Recipe with Roasted Bison and Roasted Venison (dry)

5B = Taste of the Wild Wetlands Canine Recipe with Roasted Fowl (dry)

5C = Taste of the Wild Pacific Stream Canine Recipe with Roasted Salmon (dry)

5D = Taste of the Wild Sierra Mountain Canine Recipe with Roasted Lamb (dry)

6A = Fromm Weight Management Gold (dry)

6B = Fromm Adult Gold (dry)

6C = Fromm Salmon a La Veg Recipe (dry)

6D = Fromm Lamb and Lentil Recipe (dry)

7A = Sport Dog Food Elite Grain Free Chicken Meal 30/14 (dry)

7B = Sport Dog Food Elite Grain Free Whitefish Meal 30/14 (dry)

7C = Sport Dog Food Working Dog–Grain and Peas Free Turkey Formula (dry)

8A = Nutri Source Pure Vita Duck and Oatmeal (dry)

8B = Nutri Source Pure Vita Salmon Entrée (canned)

8C = Nutri Source Weight Management Dog Food Chicken and Chicken Meal Protein (dry)

9A = Sojos Complete Turkey Recipe (raw)

10A = Stella and Chewy’s Freeze Dried Dinner Patties, Lamb and Venison Flavors (raw)

11A = Oma’s Pride Beef and Veggies (raw)

11B = Oma’s Pride Turkey and Veggies (raw)

12A = Annamaet Ultra Chicken Meal and Brown Rice (dry)

13A = Darwin’s Natural Selections Beef, Turkey and Pork (raw)

14A = Raw Bistro Dog Fare Grass-Fed Beef Entrée

15A = Earthborn Holistic Coastal Catch (dry)

16A = Natural Balance L.I.D. Limited Ingredient Diets Grain Free Potato and Duck Dry Dog Food Formula

16B = Natural Balance L.I.D. Limited Ingredient Diets Grain Free Sweet Potato and Fish Dry Dog Food Formula

16C = Natural Balance L.I.D. Limited Ingredient Diets Grain Free Chicken and Sweet Potato Canned Dog Formula

16D = Natural Balance L.I.D. Limited Ingredient Diets Grain Free Bison and Sweet Potato Dry Dog Food Formula

17A = Victor Grain Free Yukon River Canine (dry)

18A = Merrick Grain Free Wilderness Blend in Gravy (canned)

18B = Merrick Chunky Grain Free Big Texas Steak Tips Dinner in Gravy (canned)

19A = Kirkland Signature Nature’s Domain Salmon Meal and Sweet Potato Dog Food (dry)

19B = Kirkland Signature Nature’s Domain Beef Meal and Sweet Potato Dog Food (dry)

19C = Kirkland Signature Nature’s Domain Turkey Meal and Sweet Potato Dog Food (dry)

19D = Kirkland Signature Adult Formula Chicken, Rice and Vegetable Dog Food (dry)

19E = Kirkland Signature Nature’s Domain Turkey and Pea Stew for Dogs (canned)

20A = Top Quality Dog Food Beef HVM (raw)

20B = Top Quality Dog Food Chicken HVM (raw)

20C = Top Quality Dog Food Pork HVM (raw)

20D = Top Quality Dog Food Green Tripe Ground (raw)

20E = Top Quality Dog Food Green Tripe Chunks (raw)

20F = Top Quality Dog Food Beef with Tripe and Organ Meats (raw)

21A = K-9 Kravings Beef and Vegetables (raw)

21B = K-9 Kravings Chicken, Beef and Vegetables (raw)

22A = Canidae Grain Free Pure Ancestral Fish Formula Raw Coated Dry Dog Food

23A = Orijen Original Dog Food (dry)

23B = Orijen Tundra Dog Food (dry)

24A = Blue Buffalo Life Protection Formula Lamb and Brown Rice Recipe (dry)

25A = Primal Freeze-Dried Nuggets, Beef and Duck Flavor (raw)

26A = Wellness Core Grain Free Large Breed (dry)

27A = Zignature Lamb Formula (dry)

The authors commented that food 24A (Table 2) was included in the Non-traditionally fed animal group. This was because this diet was one of multiple diets fed to a single animal and comprised only a small portion of that animal’s daily calorie intake which was otherwise correctly entirely classified as non-traditional; the authors noted that removal of this dog from the analyses does not alter the study conclusions.
4. The consulted Academic Editor disagreed with the decision to exclude soy from the legume group when categorizing diets.
  The authors agreed that soy is a legume, but they stated that its exclusion from the legume group was necessary to align the study design with ongoing work by the FDA. The FDA has reported that peas and lentils are two of the most associated ingredients in patients reported to have nutritionally mediated DCM [3]. These findings have since been independently replicated and the association in the publicly available FDA data set has continued to strengthen [3].
  The authors and consulted experts disagreed about this aspect of the study design, but the *PLOS ONE* Editors acknowledge that the exclusion of soy from the legume category was clearly disclosed in the article. To further address this issue, the updated conclusion statement provided above in point 1 specifies ‘non-soy legumes’. This study did not include analyses to specifically evaluate the impacts of soy products, nor did it include subgroup analyses to examine how results were affected by inclusion of non-soy legumes vs. potatoes in the non-traditional diets.
5. Concerns were raised about food intake data. Dogs were fed fewer calories than indicated by the MER calculations, and they could be on multiple diets and/or ingest food and other supplements other than the indicated diets. Furthermore, food intake was not regulated or consistent within or across groups, and actual vs. MER data are missing for 17/43 non-traditional (but 0/43 traditional) diet animals. For those 17 animals, the table legend indicates that either food intake data were missing or dogs received most calories from other food sources; these two issues may have notably different implications when considering animal diet and outcome data.
  The authors noted that these issues stem from the limitations of the observational study design, and the quality of data that were available to the researchers. S1 File with this notice reports additional analyses excluding dogs for which MER data are not available.
6. Concerns were raised about the validity of averaging values across diets to estimate the difference in actual vs. MER for dogs who were fed multiple diets, particularly considering there may be differences in the amount of time of the different diets.
  The authors noted that averaging the values within diet groups is meant to produce an estimate of the outcome from the predictor of interest (diet group) when the confounding variables’ effects are averaged out. While the multiple diets were generally too varied to allow for control of those different diet subtypes, the authors took this approach in an attempt to use the available MER information to control for that variability by including it in analyses of outcomes.
  In follow-up to this issue, and to reexamine effects of different factors, the data were analyzed using logistic regression models fit to model the tendency of abnormal values in tau; fractional shortening; LVIDd; and LVIDs by diet, age, body condition, weight and active RER deficit (RER active/sedentary are highly collinear so only one was used, active was selected arbitrarily). Backward selection via the Akaike information criteria was then applied. Data were subset to only include patients with RER data.
  These analyses indicated that abnormal tau values are not meaningfully related to diet (by backward selection) and that the remaining effects were from active RER deficit (p = 0.038) and weight (p = 0.027).
  For fractional shortening, age (p = 0.043), weight (p = 0.152), and diet group survived model selection, but the complete separation in the diet group prevents an estimate of its effect from logistic regression (0 of 43 traditional-diet patients were abnormal versus 6 of 26 in the non-traditional group). Fisher’s exact test was applied to diet versus fractional shortening abnormality to determine the degree of relationship; this analysis did not examine effects of age. A significant difference was observed in the rate of abnormal fractional shortening with traditional-diet patients being significantly less likely to have fractional shortening abnormalities. (95% CI from 0 to 0.4, p = 0.002).
  For LVIDd, diet and weight (p = 0.135) were the remaining effects post-selection with complete separation by diet (0 of 43 traditional were abnormal versus 5 of 26 non-traditional). The Fisher’s exact test found a significant relationship (95% CI from 0 to 0.4, p = 0.006).
  For consideration of abnormal LVIDs while controlling for other factors, backward selection retained diet group (p = 0.01), age (p = 0.008), and weight (p = 0.12). The TD group was significantly less likely to experience abnormal LVIDs (OR 0.03, 95% CI 0.003–0.45).
7. The article did not discuss how authors ensured that the selected sample size provided sufficient power for the outcomes of interest.
  The authors clarified that a power calculation for this study was based upon a primary study variable (fractional shortening) and used preliminary breed-specific data. It indicated that at least 35 dogs should be used for each group to see what the authors recognize to be a clinically meaningful change in fractional shortening of 15% (approximately an absolute reduction from mean of 5 points) with a 90% power. Indeed, a significant difference in this variable was observed with the numbers recruited for this study.
8. Concerns were raised about two uses of the Fisher’s exact test. (i) This test was used to analyze whole blood and plasma taurine levels. The test assumes that the observations are independent, which would not be the case when whole blood and plasma taurine levels from the same animal are included in the analysis. (ii) The test was used to compare between groups the presence of abnormal levels of taurine or echocardiographic measurements, but when presenting the RR, 95% CI, and corresponding p-values, it appears as though the approach used for CI and p-value calculations was based on the normal approximation. Given the small numbers, the normal approximation is likely not valid.
  In response, the authors reviewed and altered this assessment due to the patients each having two observations included in the test. The Fisher’s exact test was reapplied such that each patient was included only once. This was done by having abnormal taurine summarized as having abnormal taurine in whole blood and/or plasma. This value was then tabulated against diet as traditional or nontraditional. The correct p-value for taurine by group is p = 0.0229 with an odds ratio of estimate of patients with non-traditional diets being 9.26 times (95% confidence interval from 1.15 to 215.39) as likely to have abnormal taurine in their whole blood and/or plasma. Seven non-traditional diet patients had abnormal taurine concentrations while 31 did not. Only 1 traditional diet patient had an abnormal taurine concentration while 42 did not.

The authors stated that the major findings of the original manuscript are upheld by the additional statistical analyses reported in points 6 and 8, above, and in S1 File. Non-traditional diet, as defined in the study, was significantly associated with findings that support development of nutritionally-mediated dilated cardiomyopathy. These findings continue to support the need for additional research into nutritionally mediated dilated cardiomyopathy in dogs and continue to mirror the plethora of evidence published since the time this work was completed.

A statistical reviewer evaluated the analyses reported in points 6–8 and advised that the statistical methods used were appropriate and the outcomes supported the results reported in the article, with the updated conclusions discussed above in point 1.

## Supporting information

S1 File Analyses excluding dogs for which MER data are not available. (DOCX)
