# Supplementary figures and images for: Development of plasma and whole blood taurine reference ranges and identification of dietary features associated with taurine deficiency and dilated cardiomyopathy in golden retrievers: A prospective, observational study
Source: PLoS One. 2020 May 15;15(5):e0233206. doi: 10.1371/journal.pone.0233206 (PMC7228784; doi:10.1371/journal.pone.0233206)

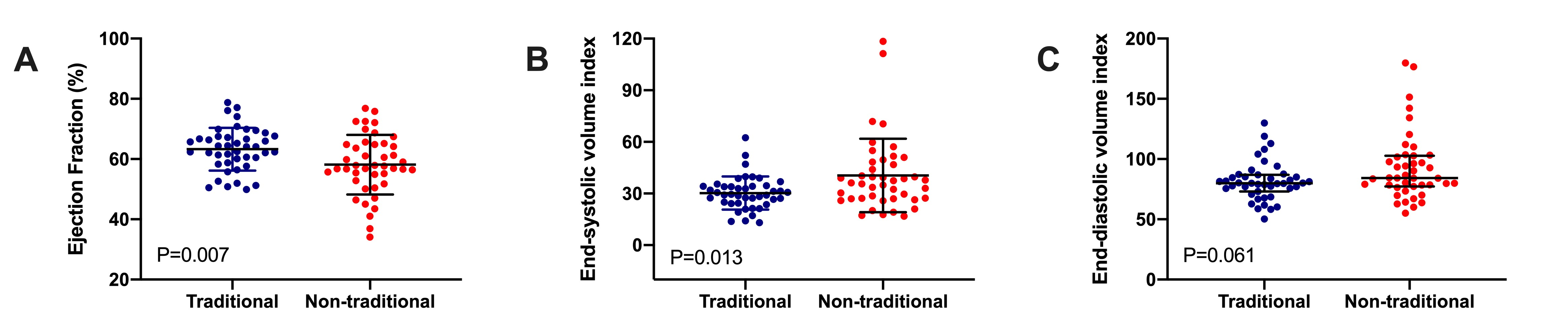

Supplement: S1 Fig — A) Unpaired t-test results for ejection fraction percent for different diet groups. B) Mann-Whitney test results for end-systolic volume index for different diet groups. C) Mann-Whitney test results for end-diastolic volume index for different diet groups. (TIF) [file pone.0233206.s001.tif]
