# Supplementary material for: Correction: Development of plasma and whole blood taurine reference ranges and identification of dietary features associated with taurine deficiency and dilated cardiomyopathy in golden retrievers: a prospective, observational study
Source: PLoS One. 2023 Aug 31;18(8):e0291101. doi: 10.1371/journal.pone.0291101 (PMC10470894; doi:10.1371/journal.pone.0291101)
Supplement: S1 File — (DOCX) [file pone.0291101.s001.docx]

**Analyses excluding dogs for which differences between fed kcal and MER data are not available**

*These analyses included n=43 and n=26 dogs in the TD and NTD groups, respectively. Four results (indicated by * below) differed in these analyses as compared to the analyses reported in the original article for which dogs without actual fed kcal to MER difference data were included.*

The mean age +/- SD of the dogs was 3.2 +/- 3.2 and 4.2 +/- 3.1 years respectively for the TD and NTD groups (p=0.99).

Body weight (mean +/- SD) was 28.9 +/- 4.9 and 32 +/- 4.4 for the TD and NTD groups respectively and was significantly higher in the NTD group (p=0.04).

Body condition score had a median of 5 (IQR 5,6) in each group (p=0.41).

*Whole blood taurine concentrations were not significantly different with mean +/- SD of 296.8 +/- 47.4 and 268.5 +/- 83.5 for the TD and NTD groups respectively (p=0.11). By comparison, whole blood taurine concentrations were found to be significantly lower in the NTD group (p.01) in the analyses reported in the original article. This change in results likely represents the reduced sample size and should be evaluated in future, larger, studies.

Plasma blood taurine concentrations were not significantly different between groups with means of 106 +/- 36.2 in the TD group and 103.9 +/- 37.9 in the NTD group (p=0.83).

Fractional shortening was lowered in the NTD group (29.1 +/- 6.7%; range 13.1 - 46%) compared to the TD group (34.2 +/- 5.2%; range 25.3-46.8%; p=0.001).

Left ventricular diameter in diastole was significantly increased in the NTD group (median 45.3, IQR 42, 48.85, range 37-62.2) compared to the TD group (median 41.1, IQR 39.3, 43.8, range 34.3-49.3; p<0.001).

Left ventricular diameter in systole was significantly increased in the NTD group (median 31.3, IQR 29, 36.2, range 22-51.7) compared to the TD group (median 27.5, IQR 25.3,29.4, range 19.9-36; p<0.001).

*There was no significant difference in the rates of low taurine with diet (p=0.06) with 4 instances of low taurine in the NTD group and 1 in the TD group of 26 and 43 patients respectively. By contrast, the analyses reported in the original article found that “*total instances of low taurine (either whole blood or plasma) were associated with feeding a NTD diet (P = 0.006).*” This difference in results likely represents the reduced sample size and should be evaluated in future, larger, studies.

Fisher's exact testing identified low FS% was associated with feeding a NTD diet (p=0.002). There were 6 instances of low FS% in the NTD group and 0 instances in the TD group of 26 and 43 patients respectively.

Fisher's exact testing identified that an elevated LVIDs was associated with the NTD diet group (p=0.001). There were 8 instances of increased LVIDs in the NTD group and 1 instance in the TD group of 26 and 43 patients respectively.

*Fisher's exact testing revealed a significant association between diet group and increased LVIDd (p=0.006). There were 5 instances of elevated LVIDd measures in the NTD group and 0 instances of elevated LVIDd measures in the TD group of 26 and 43 patients respectively. By contrast, the result reported in the original article for the full group was: “*Fisher’s exact testing did not reveal a significant association between diet group and increased LVIDd (P = 0.06)*.”

For the 68 dogs where both whole blood and plasma taurine concentrations were available, Spearman correlation evaluation revealed a significant moderate correlation (P<0.0001; r=0.53, r^2=0.28).

The median percent difference between the number of calories fed and the calculated sedentary or active energy requirements was -19.88% (IQR -33.0, -12.3) and -29.9 (IQR -42.2, -1.7) respectively for the TD group. The median percent difference between the number of calories fed and the calculated sedentary or active energy requirements was -21.93% (IQR -32.98, -14.0) and -31.69 (IQR -41.4, -24.8) respectively for the NTD group. No significant difference was identified when comparing the TD to the NTD group for either sedentary (p=0.22) or active (p=0.23) RER calculations.

End-systolic volume index was significantly higher in the NTD group (median 39.49, IQR 33.0, 51.6, range 17.75–118.4) compared to the TD group (median 29.0, IQR 24.0, 34.0, range 13.1–62.4; P = 0.0001).

Ejection fraction was significantly lower in the NTD group (55 +/- 9.8%; range 34–77%) compared to the TD group (64.07 +/- 6.8%; range 50–79%; P <0.0001)

*End-diastolic volume index was significantly different between the NTD group (median 91.49, IQR 80.01, 103.1, range 60.1–179.8) when compared to the TD group (median 79.7, IQR 69.7, 85.1, range 50.3–129.9; P = 0.0012. This represents newly identified significance when dogs without MER data were excluded.

The median number of months a dog was fed a diet was shorter in the TD group with a median of 7 (IQR 5.0, 21.0) compared to the NTD group with a median of 15 (IQR 8.0, 3.0; P = 0.03).
